# Network meta-analysis and pharmacoeconomic evaluation of antibiotics for the treatment of patients infected with complicated skin and soft structure infection and hospital-acquired or ventilator-associated penumonia

**DOI:** 10.1186/s13756-019-0518-2

**Published:** 2019-05-06

**Authors:** Ying Zhang, Yan Wang, Mieke L. Van Driel, Treasure M. McGuire, Tao Zhang, Yuzhu Dong, Yang Liu, Leichao Liu, Ruifang Hao, Lu Cao, Jianfeng Xing, Yalin Dong

**Affiliations:** 1 Department of Pharmacy, The First Affiliated Hospital of Xi’an Jiaotong University, Xi’an, 710061 China; 2 School of Pharmacy, University of Queensland, Brisbane, Queensland Australia; 3 Primary Care Clinical Unit, Faculty of Medicine, University of Queensland, Brisbane, Queensland Australia; 4 Faculty of Health Sciences & Medicine, Bond University, Gold Coast, Queensland Australia; 5 Mater Pharmacy Services, Mater Health Services, Brisbane, Queensland Australia; 6 Department of Pharmacy, Xi’an Jiaotong University, Xi’an, 710061 China

## Abstract

**Background:**

Infections due to methicillin-resistant *Staphylococcus aureus (*MRSA) cause serious health risks and significant economic burdens and the preferred drugs are still controversial.

**Methods:**

We performed a network meta-analysis (NMA) to compare the efficacy and safety of antibiotics used to treat inpatients with complicated skin and soft structure infections (cSSSI) or hospital-acquired or ventilator-associated pneumonia (HAP/VAP). We also developed a decision tree model to assess the cost-effectiveness of antibiotics.

**Results:**

Forty-nine randomized controlled trials met the inclusion criteria (34 for cSSSI, 15 for HAP/VAP) and compared the efficacy and safety of 16 antibiotics. For cSSSI, NMA indicated that for clinical cure, linezolid was superior than vancomycin (odds ratio (OR) 1.55, 95% confidence interval (CI) 1.19–2.02), while tedizolid (OR 1.39, CI 0.70–2.76) was similar to vancomycin. In terms of safety, there were no significant differences between any two interventions on total adverse events. Based on drug and hospital costs in America, the incremental cost-effectiveness ratios (ICERs) per life-year saved for linezolid and tedizolid compared with vancomycin were US$2833 and US$5523. For HAP/VAP, there were no significant effects either for clinical cure or for safety endpoints between linezolid and vancomycin in NMA. ICERs per life-year saved for linezolid compared with vancomycin were US$2185.

**Conclusion:**

In these clinical trials, considering efficacy, safety, and cost-effectivenes, linezolid and tedizolid showed their superiority in MRSA cSSSI; while linezolid might be recommended to treat MRSA pneumonia. Although vancomycin was not cost-effective in pharmacoeconomic evaluation, it is still the first-line treatment for MRSA infection in the clinical practice. This study might provide new insights of therapeutic choices for patients with MRSA infections whilst awaiting the arrival of higher quality evidence.

**Electronic supplementary material:**

The online version of this article (10.1186/s13756-019-0518-2) contains supplementary material, which is available to authorized users.

## Introduction

Methicillin-resistant *Staphylococcus aureus* (MRSA) infections have posed a global threat since the end of the last century. MRSA infections cause increased mortality, cost burden and longer hospital stay [1]. Resistance tracking by SENTRY highlights the increasing prevalence of MRSA for two infections in which *S. aureus* predominates: complicated skin and soft-structure infections (cSSSIs); and hospital-acquired pneumonia (HAP) or ventilator-associated pneumonia (VAP) [2, 3].

The Infectious Diseases Society of America (IDSA) [4] published clinical practice guidelines for the treatment of MRSA infections, providing a practical basis for management. The glycopeptide vancomycin has been recommended by IDSA guidelines of MRSA treatment for many decades due to its excellent antibacterial activity [5]. However, in the latest decade, minimum inhibitory concentration (MIC) ‘creep’ among susceptible strains to vancomycin has been observed among MRSA isolates in USA, this has been consistently associated with increased mortality [6, 7].

In addition, there has recently been a sharp rise in the incidence of infections caused by MRSA [8, 9]. This has resulted in increased use of vancomycin and the emergence of vancomycin resistant *S. aureus* [9, 10]. As a consequence, there has been an emergence of new antibiotics to combat the evolving resistance of this challenging pathogen [11]. Linezolid, daptomycin, tigecycline, new glycopeptides such as telavancin and ceftaroline have been approved to treat MRSA infections. While meta-analyses [12–15] which have compared the efficacy and safety of vancomycin with linezolid or other antibiotics has been published; the results of these studies are not consistent and definitive conclusions cannot be drawn.

In this paper, we compared different antibiotics used for the treatment of MRSA infections using the technique of network meta-analysis (NMA) to determine which antibiotic(s) achieve the superior therapeutic effect. This approach can simultaneously assess multiple interventions in combination with direct and indirect evidence, thereby synthesizing available evidence. In addition, we developed a pharmacoeconomic analysis model from the patient perspective to investigate the cost-effectiveness of these antibiotics using clinical efficacy data generated by the NMA and medical data.

## Materials and methods

### Search strategy and selection criteria

A network meta-analysis of randomized controlled trials (RCTs) was performed in compliance with the Preferred Reporting Items for Systematic Reviews and Meta-analyses (PRISMA) guidelines [16]. We searched PubMed, EMBASE, and the Cochrane Central Register of Controlled Trials for RCTs published up to September 2018, using the search strategies described in Additional file 1: Appendix A. We also searched ClinicalTrials.gov for any relevant completed studies and hand searched the references from the retrieved RCTs.

### Study selection and data extraction

Infections qualifying for this research were cSSSIs [17] and HAP/VAP in adult patients [18, 19]. However, most of the studies we included were published prior to the Food and Drug Administration (FDA) guidance on designing RCTs to evaluate drugs for cSSSIs [20], with the results that the definition of cSSSI varied between studies. Therefore, we did not present a clear definition to avoid misunderstanding. HAP is defined as pneumonia that occurs 48 h or more after admission, which was not incubating at admission [21, 22]. VAP refers to pneumonia that occurs within 48–72 h after endotracheal intubation [21, 23]. To minimise clinical heterogeneity and because it is difficult to predict to what extent the increased prevalence of community-associated MRSA (CA-MRSA) will be accompanied by enhanced toxicity [24–27], we did not evaluate CA-MRSA infections.

The following eligibility criteria were applied: RCTs including adult patients with MRSA-related infections, and involving antibiotics with anti-MRSA activity. Studies were excluded if: (1) non-English language, (2) involving colonization or infection prevention, (3) pharmacokinetic/pharmacodynamic studies, (4) the research only evaluated pharmacoeconomics or pooled analysis. Two researchers (YZ and YW) independently assessed the citations against the above eligibility criteria. Disagreements about study selection and data extraction were resolved through consensus.

The following data were extracted: authors, year of publication, patient population, study design, baseline characteristics, interventions (antibiotics for MRSA infections), clinical outcomes and sponsorship.

### Outcomes and quality assessment

The primary outcome was clinical success at test of cure (TOC) in the modified intention-to-treat (mITT) population, which was determined cured and improved. Cured was defined as resolution of the clinical signs and symptoms of infection when compared with baseline; improved was defined as improvement in two or more, but not all, clinical signs and symptoms of infection when compared with baseline. The intent-to-treat (ITT) population included all patients randomized into the study. The mITT population was randomized patients receiving at least one dose of the study drug. The secondary endpoints included clinical efficacy in the clinically evaluable (CE) population and microbiological eradication in the microbiologically evaluable (ME) population. We also evaluated overall and serious adverse events and all-cause mortality in the safety population as safety outcomes. Since the definitions of mortality were different in each trial, we did not give specific time frame of mortality. The safety population was the primary population for all the safety analyses, and consisted of all patients who were dosed with study drug, irrespective of randomization. Since the included RCTs used different definitions for adverse events, such as nephrotoxicity and thrombocytopenia, the safety was assessed according to each RCT’s own definition.

We evaluated the quality of included RCTs using the Cochrane risk of bias assessment tool in Review Manager version 5.3.

### Pharmacoeconomic analysis model

We conducted a decision-analytic model from the patient perspective to assess the outcomes of antibiotic therapy with antibiotics in patients with MRSA infections using TreeAge Pro 2011 (TreeAge Software, Inc., MA, USA). The decision tree model followed first-line and second-line therapy for MRSA cSSSI or HAP/VAP (see Additional file 1: Figure B.1).

Vancomycin was considered as comparator in the MRSA cSSSI model and the pneumonia model. The cure rate of the first-line treatment for the comparator was extracted from the pooled data of pairwise meta-analysis. The ORs of clinical cure for the comparator versus each antibiotic were obtained from NMA to generate the probabilities for comparator agents. The key outcomes of lifetime costs and life-years (LYs) saved for the two types of MRSA infections. The incremental cost-effectiveness ratios (ICERs) per LY saved was calculated, which was used to compare the performance of treatment strategies. We did not adjust for quality of life because we assumed that survivors of MRSA infections are unlikely to have long-term consequences related to this condition (i.e., utility value = 1) [28]. We considered each treatment strategy resulting in an effect size less than the willingness-to-pay (WTP) threshold ($50,000) to be acceptable [29]. One-way sensitivity analyses were conducted to test how variation in one variable could affect model results. Probabilistic sensitivity analysis was carried out with 1000 times of Monte Carlo simulations to evaluate the impact of all variables simultaneously. The model and associated deterministic sensitivity analysis were developed from the patient perspective, with all costs are presented in 2017 US dollars with a conversion rate of 3% [30] (additional details of the cost-effectiveness methodology in Additional file 1: Appendix B).

### Statistical analysis

We conducted two types of meta-analyses. First, we performed pairwise meta-analysis with a random-effects model. The estimates of primary and secondary outcomes were determined using odds ratios (OR) and their corresponding 95% confidence intervals (CI). Secondly, we conducted a random-effects network meta-analysis for direct and indirect comparisons [31]**.** In order to estimate the rank order for all interventions, surface under the cumulative ranking curve (SUCRA) probabilities were reported for primary outcome and safety outcome [32]. To check for the presence of inconsistency, we used the transitivity assumption to assess consistency of our assumption by comparing direct and indirect overall effects [33]. A common estimate (the tau [τ] value) was assumed to evaluate the heterogeneity for all comparisons. To examine the robustness of the estimates with different baseline characteristics, sensitivity network meta-analyses for primary outcomes were performed on the following variables: sex ratio, age and restricting to low-risk of bias studies. We used Stata (Version 13.0) for the analyses (additional details of the statistical analysis of meta-analysis in Additional file 1: Appendix C).

## Results

### Characteristics of included trials

**Fig. 1:**
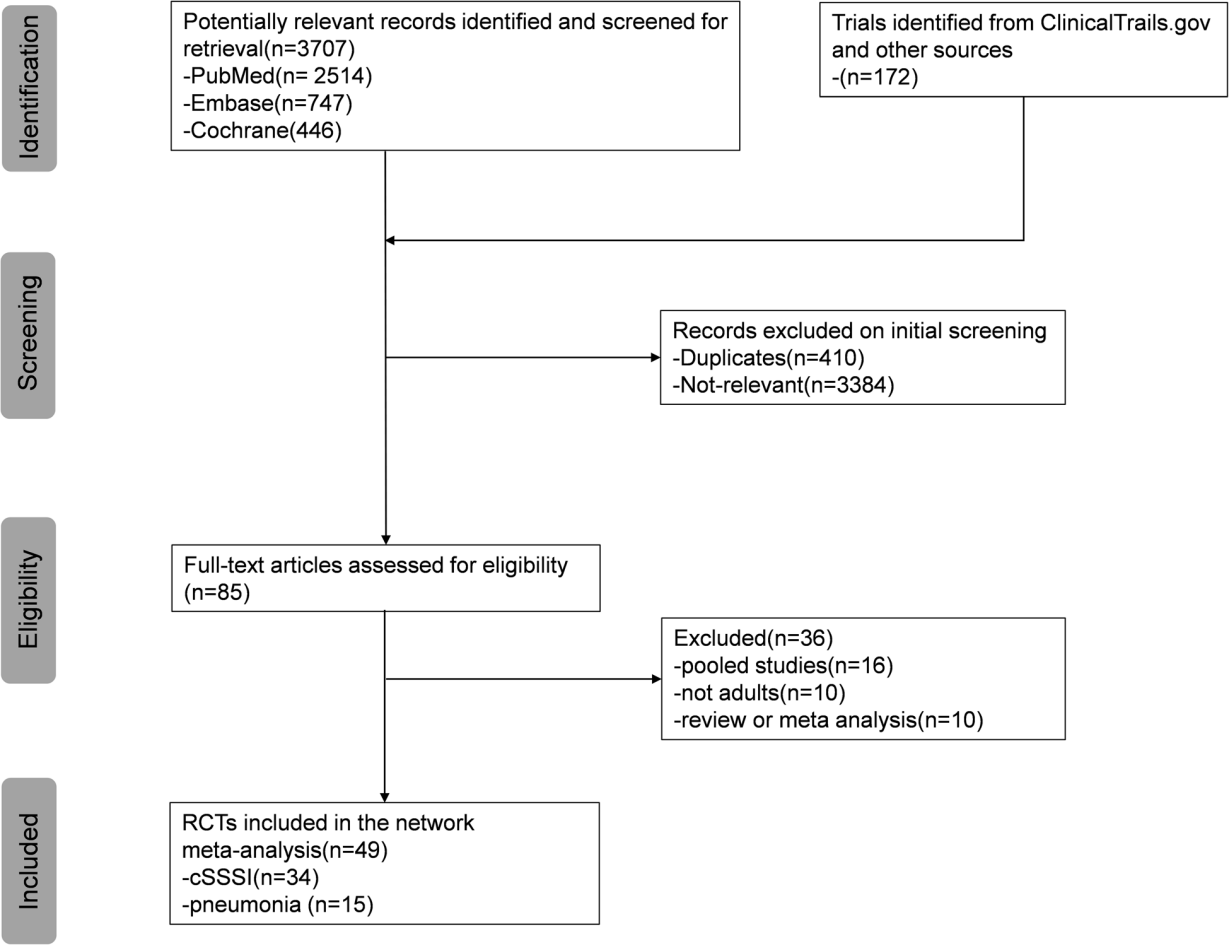
Study selection flow diagram. RCT, randomized controlled trial. cSSSI, complicated skin and soft structure infection

**Fig. 2:**
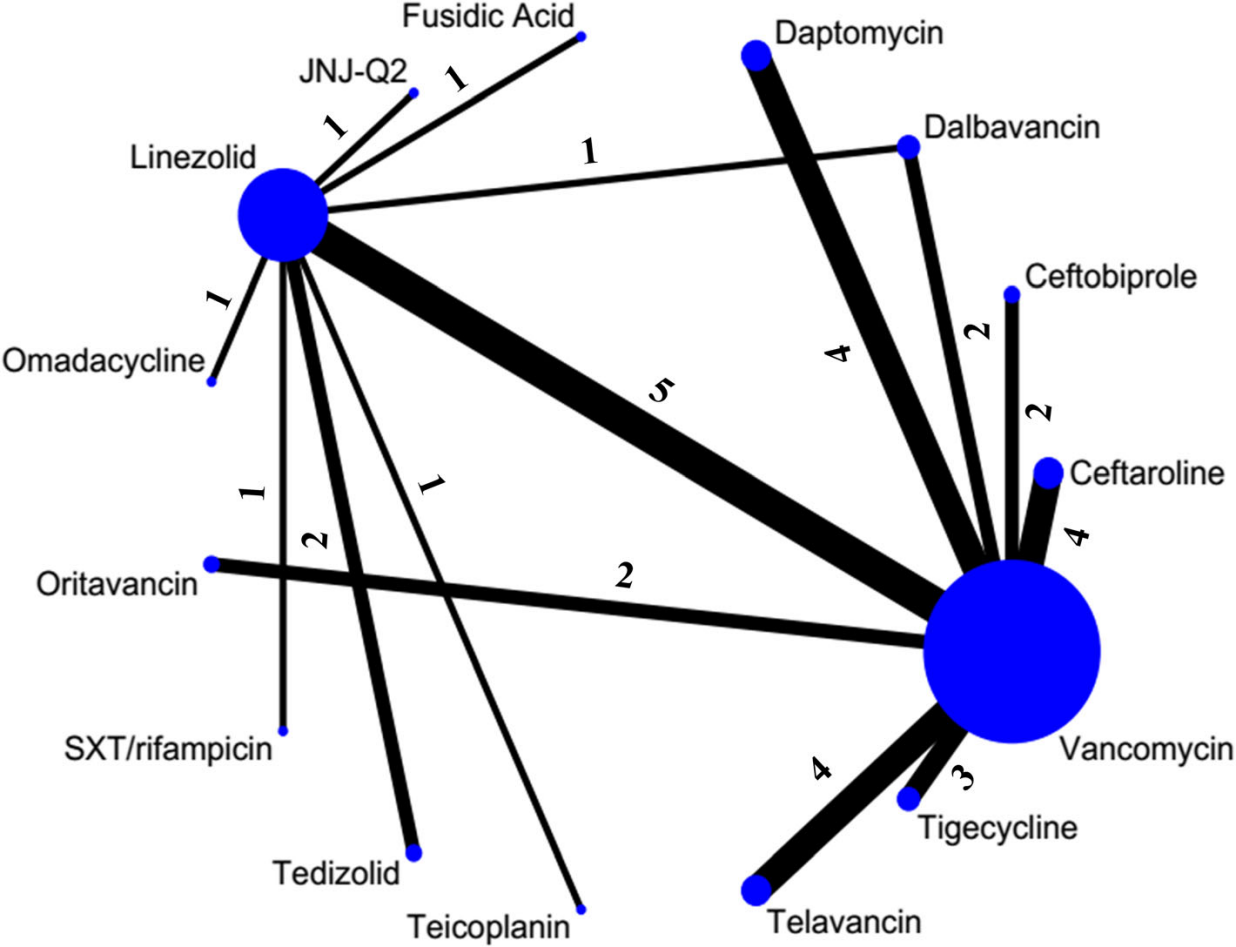
Network plot of eligible comparisons for primary outcome in cSSSI group. The size of the node corresponds to the number of individual studies. The directly compared interventions are linked with a line, the width of which is proportional to the number of studies assessing respective comparisons. Numbers above the lines indicate studies. SXT, trimethoprim/sulfamethoxazole. JNJ-Q2, a novel fluoroquinolone

**Fig. 3:**
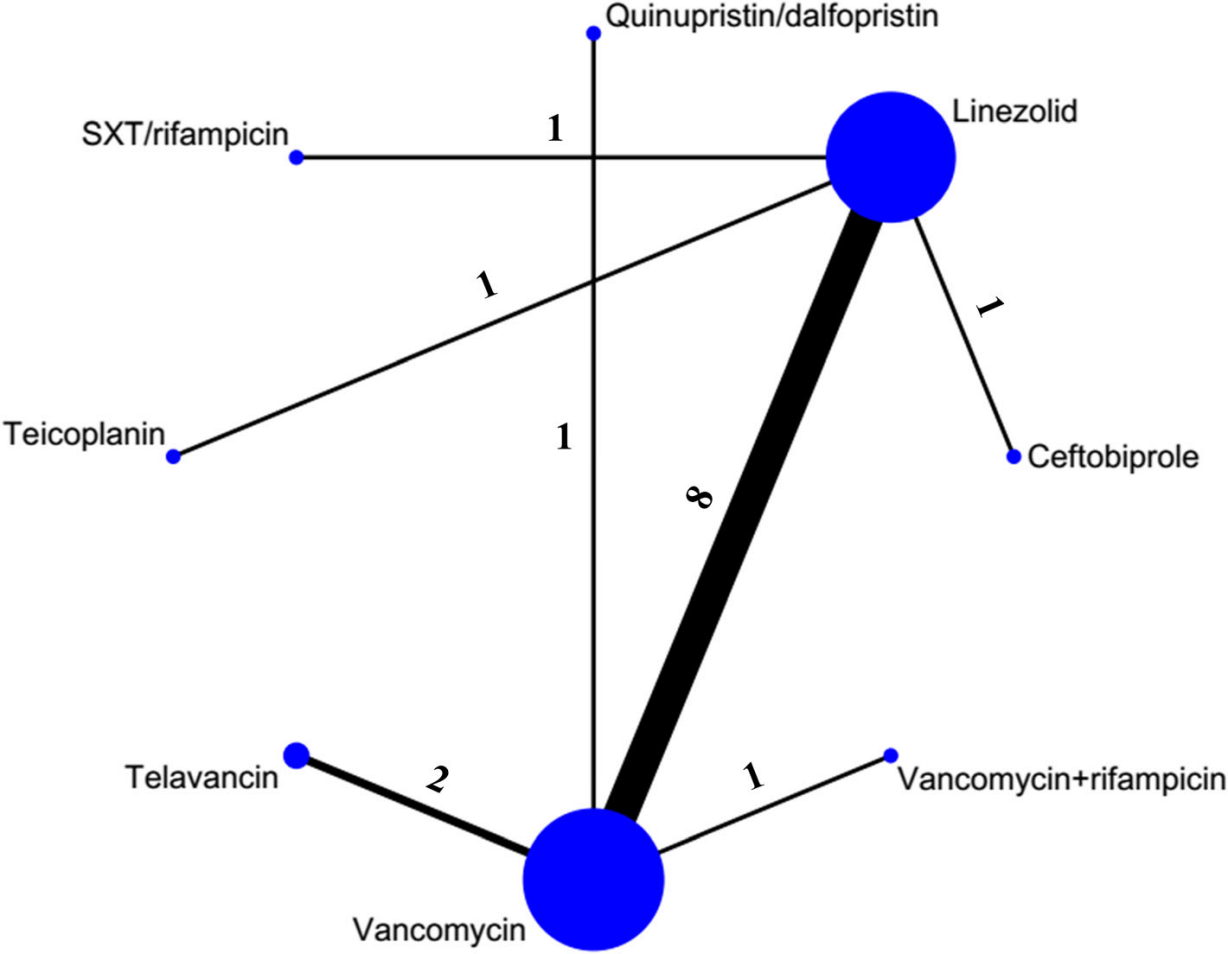
Network plot of eligible comparisons for primary outcome in HAP/VAP group. The size of the node corresponds to the number of individual studies. The directly compared interventions are linked with a line, the width of which is proportional to the number of studies assessing respective comparisons. Numbers above the lines indicate studies. SXT, trimethoprim/sulfamethoxazole

Of 3879 potentially relevant articles identified through the literature search, 3794 were excluded after the initial screening (Fig. 1). We reviewed the full text of the remaining 85 studies. We excluded 36 studies for the following reasons: pooled studies (*n* = 16), non-adult patients (*n* = 10), reviews or meta-analyses (n = 10). Finally, 49 trials studying 16 different antibiotics met the criteria (34 for cSSSI, 15 for pneumonia) and were included in the NMA. The main characteristics of the included studies are shown in Additional file 1: Appendix D. The eligible comparison networks with MRSA cSSSI and pneumonia respectively are shown in Figs. 2 and 3.

The risk of bias summary and graph are listed in Additional file 1: Appendix E. Some studies were assessed as unclear risk of bias as they did not provide details about randomization, allocation concealment and blinding. The majority of studies are randomized, double-blind, controlled trials. Six studies reported evidence of blinding of participants and personnel. Most of the studies were sponsored by pharmaceutical companies (42 RCTs, 87%).

### Pairwise and network meta-analysis of cSSSI

**Fig. 4:**
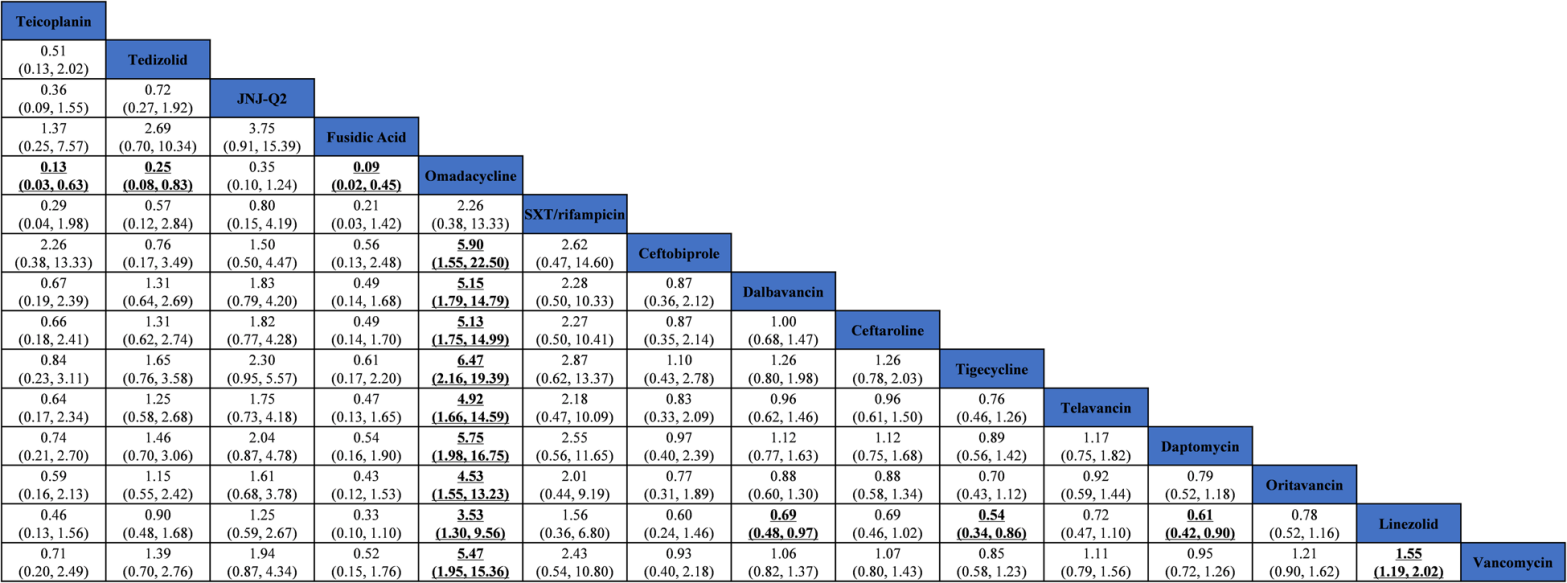
Network meta-analysis of clinical cure in cSSSI in mITT population. Comparisons should be read from left to right. The safety estimate is located at the intersection of the column-defining treatment and the row-defining treatment. Significant results are in bold and underlined. cSSSI, complicated skin and soft structure infection. mITT, modified intention-to-treat population. JNJ-Q2, a novel fluoroquinolone. VAN, vancomycin. LIN, linezolid. CEF^1^, ceftaroline. CEF^2^, ceftobiprole. SXT/RIT, trimethoprim/sulfamethoxazole plus rifampicin. ORI, oritavancin. DAL, dalbavancin. DAP, daptomycin. TEL, telavancin. TIG, tigecyline. TEI, teicoplanin. OMA, omadacycline. FA, fusidic acid. JNJ-Q2, a novel fluoroquinolone. TED, tedizolid. OR, odds ratio. CI, confidence interval

For cSSSI, all 34 included studies reported clinical cure rate in the mITT population. Pairwise meta-analysis showed that linezolid was more effective than vancomycin (OR 1.23, 95% CI 1.09–1.50, *I*^*2*^ = 0.0%) (Table 1), which was consistent with the results of the NMA (OR 1.55, 95% CI 1.19–2.02) (Fig. 4). Moreover, among patients with MRSA cSSSI, omadacycline was found to have a superior clinical response compared to all antibiotics except trimethoprim/sulfamethoxazole plus rifampicin (SXT/rifampin) (Fig. 4). Only 14 studies assessed the microbiological eradication of the ME population. Pairwise meta-analysis showed that linezolid was significantly superior to vancomycin (OR 1.93, 95% CI 1.26–2.96) in the microbiological response, which was consistent with the results of NMA (OR 2.42, 95% CI 1.21–4.86) (see Additional file 1: Table F.1 and Figure F.1).

**Table 1:** Pairwise meta-analysis of antibiotics of clinical cure in cSSSI and HAP/VAP in mITT population

Treatment comparisons	Number of studies	Pairwise meta-analysis Odds ratio (95% CI)	*P* value	Heterogeneity *I*^*2*^, % (variation in OR attributable to heterogeneity)
cSSSI				
Vancomycin vs Oritavancin	2	0.96 (0.59, 1.56)	0.867	0.0
Vancomycin vs Daptomycin	4	1.04 (0.79, 1.36)	0.756	0.0
Vancomycin vs Telavancin	4	0.86 (0.61, 1.24)	0.430	0.0
Vancomycin vs Tigecycline	3	1.15 (0.79, 1.66)	0.451	0.0
Vancomycin vs Ceftaroline	4	0.94 (0.71, 1.26)	0.695	0.0
Vancomycin vs Dalbavancin	2	1.00 (0.77, 1.31)	0.973	0.0
Vancomycin vs Ceftobiprole	2	0.78 (0.33, 1.82)	0.565	0.0
Linezolid vs Vancomycin	5	1.23 (1.09, 1.50)	0.006	0.0
Linezolid vs Dalbavancin	1	0.84 (0.39, 1.82)	0.673	–
Linezolid vs SXT/rifampicin	1	0.70 (0.16, 2.95)	0.627	–
Linezolid vs Omadacycline	1	0.36 (0.03, 4.18)	0.417	–
Linezolid vs Fusidic Acid	1	2.71 (0.83, 8.89)	0.099	–
Linezolid vs JNJ-Q2	1	0.95 (0.45, 1.94)	0.682	–
Linezolid vs Tedizolid	2	1.09 (0.58, 2.02)	0.780	0.0
Linezolid vs Teicoplanin	1	0.52 (0.15, 1.79)	0.234	–
HAP/VAP				
Vancomycin vs Telavancin	2	1.02 (0.85, 1.23)	0.822	0.0
Vancomycin vs Vancomycin/rifampicin	1	1.49 (0.72, 3.06)	0.278	–
Vancomycin vs Quinupristin/dalfopristin	1	1.03 (0.71, 1.51)	0.858	–
Linezolid vs Vancomycin	8	1.11 (0.91, 1.34)	0.301	0.0
Linezolid vs Ceftobiprole	1	0.53 (0.17, 1.57)	0.251	–
Linezolid vs Teicoplanin	1	0.38 (0.07, 2.16)	0.279	–
Linezolid vs SXT/rifampicin	1	1.77 (0.25, 12.45)	0.562	–

*cSSSI* complicated skin and soft structure infection, *HAP/VAP* hospital-acquired or ventilator-associated pneumonia, *mITT* modified intention-to-treat population, *JNJ-Q2* a novel fluoroquinolone, *SXT* trimethoprim/sulfamethoxazole

The majority of studies reported data on adverse events. There were no significant differences between any two interventions in terms of all-cause mortality rates in MRSA cSSSI patients. Telavancin had a higher total number of adverse events compared to vancomycin both in pairwise (OR 1.35, 95% CI 1.14–1.60) and network meta-analyses (OR 1.25, 95% CI 1.10–1.73) (see Additional file 1: Table F.2). The incidence of nephrotoxicity was similar between linezolid and vancomycin in head-to-head comparisons. Risk for thrombocytopenia was not statistically significantly different among linezolid and others in pairwise meta-analysis. Gastrointestinal adverse events were common with all included antibiotics in the treatment of MRSA cSSSI. (see Additional file 1: Table F.2).

The efficacy and safety of 15 interventions for the treatment of MRSA cSSSI are presented via the surface under the cumulative ranking curve (SUCRA) values which display the probability of each increasing rank order for efficacy and safety (see Additional file 1: Tables F.3 and F.4). In terms of efficacy, NMA found antibiotics ranked first to fourth via SUCRA values were omadacycline, SXT/rifampin, JNJ-Q2, and oxazolidinone. In terms of safety, the lowest ranked antibiotic was telavancin.

### Pairwise and network meta-analysis of HAP/VAP

**Fig. 5:**
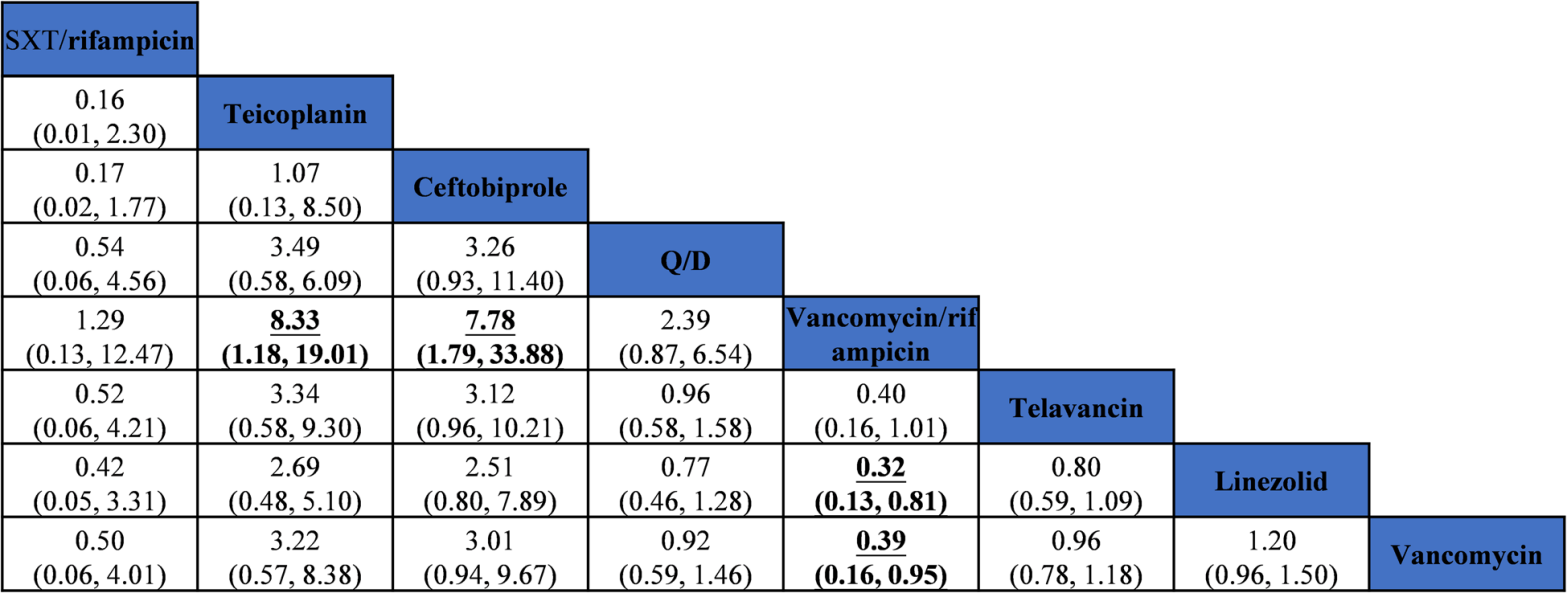
Network meta-analysis of clinical cure in pneumonia in mITT population. Comparisons should be read from left to right. The safety estimate is located at the intersection of the column-defining treatment and the row-defining treatment. Significant results are in bold and underlined. mITT, modified intention-to-treat population. VAN, vancomycin. LIN, linezolid. CEF^2^, ceftobiprole. SXT/RIT, trimethoprim/sulfamethoxazole plus rifampicin. TEL, telavancin. TEI, teicoplanin. VAN/RIF, vancomycin plus rifampicin. Q/D, quinupristin/dalfopristin OR, odds ratio. CI, confidence interval

In the smaller HAP network of studies, pairwise meta-analysis showed that for clinical cure rate linezolid was similar to vancomycin in the mITT population (OR 1.11, 95% CI 0.91–1.34, *I*^*2*^ = 0.0%) (Table 1), which was consistent with the NMA results (OR 1.20, 95% CI 0.96–1.50) (Fig. 5). In addition, the NMA of pneumonia trials found that for clinical cure rate there was no difference between teicoplanin and linezolid or teicoplanin and vancomycin (Fig. 5). The results of the head-to-head comparisons and NMA of different antibiotics in the ME population are listed in Additional file 1: Table G.1 and Figure G.1. Safety results of antibiotics are reported in Additional file 1: Table G.2. Most included studies reported on adverse events, and there was no significant difference between any two treatments for total adverse events. The incidence of nephrotoxicity of vancomycin was similar to linezolid (OR 1.02, 95% CI 0.93–1.12). The difference was also not statistically significant between vancomycin and linezolid for the incidence of thrombocytopenia (OR 0.99, 95% CI 0.86–1.55). Gastrointestinal adverse events were common with all antibiotics in the treatment of MRSA pneumonia (see Additional file 1: Table G.2).

NMA of MRSA pneumonia trials showed that ceftobiprole ranked first about efficacy, followed by teicoplanin, linezolid, and vancomycin (see Additional file 1: Table G.3). While vancomycin ranked first according to safety (see Additional file 1: Table G.4).

### Assessment of heterogeneity and inconsistency

No significant heterogeneity within pairwise comparisons of antibiotics was found (Table 1). The values of heterogeneity variables for clinical cure rate in the mITT population, microbiological response in the ME population, clinical cure rate in the CE population and the incidence of total adverse events were τ = 0.09, τ = 0.33 τ = 0.43, τ = 0.18, respectively (all values indicated moderate heterogeneity). In the inconsistency test only one closed loop (Dalbavancin-Linezolid-Vancomycin) was detected and the results are shown in Additional file 1: Appendix H. There were no substantial differences between direct and indirect treatment effect estimates and therefore no evidence of inconsistency in the cSSSI network. In the MRSA pneumonia network, indirect evidence was hardly obtained in the presence of direct evidence for any interventions, hence an inconsistency test was not performed.

### Sensitivity analysis

The results of the post hoc sensitivity analysis of MRSA cSSSI and pneumonia are presented in Additional file 1: Appendix F and G. In cSSSI, linezolid and tedizolid showed more effective than other antibiotics when high risk studies were excluded (i.e., SXT/rifampin in cSSSI). Linezolid and vancomycin were superior to other antibiotics in pneumonia after including the research with high-risk of bias (high risk studies were excluded, i.e., teicoplanin in pneumonia). The efficacy rankings were robust after adjusting for the mean age and sex ratios. The comparison-adjusted funnel plots showed no asymmetry in Additional file 1: Appendix I.

### Cost analysis

#### Base-case analysis

Ten antibiotics (vancomycin, linezolid, tedizolid, telavancin, dalbavancin, oritavancin, ceftaroline, tigecycline, daptomycin, SXT/rifampin) were included in the MRSA cSSSI model. Exclusions were fusidic acid and teicoplanin as the NMA showed they were less effective; JNJ-Q2 was not marketed; although omadacycline was approved by FDA, it was not marketed currently; and ceftobiprole has been discontinued. The costs and clinical outcomes of the 10 treatments are summarized in Additional file 1: Table B.1. Vancomycin was used as the baseline to calculate the incremental cost-effectiveness ratio (ICER) for other strategies, in accordance with current clinical guidelines [4]. Tigecycline, oritavancin, dalbavancin, telavancin, ceftaroline were less effective than vancomycin, and the ICERs per LY saved corresponded to >US$50,000. Therefore, these five options were not considered cost-effective treatment strategies. In the present analysis, SXT/rifampicin was the dominant strategy, however, there was insufficient data to recommend it since the RCT studying SXT/rifampicin was of high risk. The ICERs per LY saved for linezolid, tedizolid, daptomycin and ceftaroline relative to vancomycin corresponded to US$2833, US$5523, US$6200 and US$9057, respectively (Table 2). These five antimicrobial agents might be recommended for the treatment of MRSA cSSSI within a range of WTP thresholds.

**Table 2:** Cost-effectiveness of antibiotics for treatment of cSSSI patients

Treatment strategy	Total cost (USD)	Incremental cost (USD)	Total LY	LY saved^a^	ICER per LY saved
Vancomycin	15,254		7.52		
SXT/rifampicin	13,419	-1836	7.68	0.16	Dominant
Linezolid	16,387	1133	7.92	0.40	2833
Tedizolid	17,353	2099	7.90	0.38	5523
Daptomycin	17,238	1984	7.84	0.32	6200
Ceftaroline	17,971	2717	7.82	0.30	9057

*cSSSI* complicated skin and soft structure infection, *USD* US dollar, *LY* life-years, *ICER* incremental cost-effectiveness ratio, *SXT* trimethoprim/sulfamethoxazole

^a^Calculated as the average cost per patient and the average number of LY per patient in this strategy minus those of the treatment of vancomycin

For MRSA pneumonia, teicoplanin, vancomycin, linezolid, telavancin, SXT/rifampicin and quinupristin/dalfopristin (Q/D) were evaluated in the cost analysis. The total costs and effectiveness of the five treatment options are shown in Additional file 1: Table S2. The ICERs were calculated relative to vancomycin, which was considered the baseline for other strategies. All antibiotics including telavancin, Q/D and SXT/rifampicin were subordinate to vancomycin except linezolid and teicoplanin, since the ICERs per LY saved corresponded to >US$50,000. In the present analysis, teicoplanin was the dominant strategy, however, we were unable to recommend this treatment due to the limited data with high risk study. The ICER per LY saved for using linezolid over vancomycin was US$2185 (Table 3). These two antibiotics might be recommended for treatment of MRSA pneumonia.

**Table 3:** Cost-effectiveness of antibiotics for treatment of pneumonia patients

Treatment strategy	Total cost (USD)	Incremental cost (USD)	Total LY	LY saved^a^	ICER per LY saved
Vancomycin	16,346		9.84		
Teicoplanin	12,487	− 3858	10.80	0.96	Dominant
Linezolid	19,230	2884	11.16	1.32	2185

*USD* US dollar, *LY* life-years, *ICER* incremental cost-effectiveness ratio

^a^Calculated as the average cost per patient and the average number of LY per patient in this strategy minus those of the treatment of vancomycin

#### Sensitivity analysis

Deterministic sensitivity analyses revealed that the results were most sensitive to the costs of antibiotics included in the economic analysis and the duration of treating MRSA cSSSI and HAP/VAP. For MRSA cSSSI, the unit cost of daptomycin more than US$305 would result in more cost-effective compared with linezolid. In HAP/VAP, the unit cost of telavancin less than US$320 would make it more cost-effective than linezolid. Other variations in a single model parameter had no substantial impact on the primary analyses both in cSSSI and pneumonia.

In cSSSI, probabilistic sensitivity analyses (PSA) displayed that SXT/rifampin, linezolid, tedizolid, daptomycin, ceftaroline, tigecycline, dalbavancin, oritavancin and telavancin had probabilities of 88.7, 87.7, 87.2, 86.7, 84.1, 72.4, 69.0, 52.1, and 50.0%, respectively, of being cost-effective relative to vancomycin under the WTP threshold ($50,000). As for pneumonia, teicoplanin, linezolid, Q/D, telavancin and SXT/rifampin had probabilities of 83.2, 60.0, 34.3, 26.3, 17.5%, respectively within the threshold.

## Discussion

Our systematic review and NMA comprehensively evaluated the efficacy, safety, and cost-effectiveness of antibiotics used to treat MRSA cSSSI and pneumonia. We found that for clinical cure linezolid and tedizolid were superior to the other antibiotics in patients with cSSSI. In terms of safety, there was no significant difference between any two interventions for total adverse events. Our cost analysis showed that oxazolidinone and vancomycin were cost-effective in the treatment of MRSA infections and within a range of WTP thresholds. For MRSA pneumonia, the efficacy of linezolid and vancomycin were better than other antibiotics. As for safety, no significant difference was found between any of the treatments for total adverse events. In the cost-analysis, linezolid and vancomycin were cost-effective within a range of WTP thresholds.

cSSSIs are common infections in everyday clinical practice, resulting in high morbidity and health care cost [34]. We found that linezolid had better efficacy in patients with cSSSI, which is consistent with guideline recommendations. Our findings are consistent with a previous study [35] which highlighted that SXT/rifampicin and linezolid had similar efficacy but lower cost [36]. Nevertheless only one RCT included in our NMA directly compared SXT/rifampicin and linezolid, there is insufficient evidence to make any conclusion. A recent large meta-analysis [37] showed that linezolid was associated with a significantly higher clinical cure rate and reduced length of hospital stay compared with vancomycin for cSSSI. The improved skin penetration of linezolid and its high bioavailability may explain the improved clinical outcomes among patients receiving linezolid [38]. Similarly, linezolid may be a cost-effective alternative to vancomycin in the treatment of patients with cSSSI [39, 40], which was consistent with our analysis. Tedizolid, a novel oxazolidinone, showed comparable efficacy to linezolid in a phase 3 RCTs [41], which was consistent with our results. Omadacycline which belongs to a new class of compounds, aminomethyl-cyclines, is considered a promising drug for the treatment of severe MRSA infections [42]. RCTs found that omadacycline was more effective than linezolid; however, due to the limited number of studies in our NMA, we are unable to conclude whether omadacycline can be used as an alternative to linezolid. Although there was no significant difference between any of the treatments for total adverse events, attention should still be paid to some adverse events for antibiotics (e.g., thrombocytopenia for linezolid, nephrotoxicity for vancomycin).

Pneumonia is the second most common hospital-acquired infections in adults, contributing to inpatient mortality [43]. Previous studies compared the efficacy of linezolid and vancomycin in the treatment of MRSA pneumonia [44–47] with contradictory results. Using NMA we showed that the clinical response with linezolid is similar to vancomycin, which is consistent with guideline recommendations. Furthermore, our analysis showed that the efficacy of teicoplanin was better than the other interventions, but differences were not statistically significant. However, as only one RCT on teicoplanin was included, this remains to be further verified. Although there was no statistically significant difference among included antibiotics, we should pay attention to several adverse events for antimicrobial agents. Telavancin was approved by FDA for the treatment of hospital-acquired and ventilator-associated bacterial pneumonia caused by *Staphylococcus aureus* [48]*.* To our knowledge, telavancin has not been compared with linezolid in any clinical or observational studies. Our study compared these two drugs indirectly and found no significant differences in efficacy. Serious adverse events of telavancin can cause poor clinical outcomes among patients, which may limit its application in clinical practice. HAP caused an increase of $11,897 to $25,072 per incident and was the leading cause of death in all nosocomial infections [49]. In four pharmacoeconomic analyses comparing vancomycin and linezolid in the treatment of pneumonia, efficacy and cost data were obtained from published RCTs [50–53]. Our results confirmed the results of previous analyses and showed that despite its higher cost, linezolid was cost-effective for treatment of MRSA pneumonia.

Given the difficulties of assessing multiple RCTs to directly compare individual antibiotics used to treat severe MRSA infections, the use of NMA to compare the relative efficacy and safety of new and traditional drugs through indirect methods is recommended. Previous studies have evaluated the effectiveness and safety of antibiotics used for treating hospitalized adults with cSSSI or pneumonia [14, 15, 54]. However, timely evaluation of the latest treatment strategies in the era of antimicrobial resistance is crucial for treatment decision making.

Our study has several limitations. First, most of the research were published prior to the 2010 FDA guideline for designing RCTs to evaluate antibiotics for cSSSI [20]. As a consequence, the definition of cSSSI varied between studies. Second, the low quality of some research due to unclear bias risk and the possibility of publication bias, may jeopardize the validity of our conclusions. Findings from our analysis, however, were not affected by the results from the research with high-risk of bias via sensitivity analysis. Third, the confidence intervals for some antibiotics are really wide because of limited sample size. Therefore, we recommend that larger research should be included in order to make a more accurate conclusion. Fourth, due to various adverse events of involved antibiotics, we only describe the probability and costs associated with primary adverse events (e.g thrombocytopenia for linezolid or nephrotoxicity for vancomycin). Ignoring differences in the probability and costs of adverse events may influence results. Finally, our cost analysis only evaluated linezolid intravenous formulations. Linezolid has the main advantage of oral administration, with almost 100% bioavailability [55]. Given the high bioavailability of linezolid [56], future studies should consider the cost-effectiveness of oral dosage forms to comprehensively evaluate the clinical value of antibiotics for the treatment of MRSA infections. This study might provide new insights of therapeutic choices for patients with MRSA infections whilst awaiting the arrival of higher quality evidence.

## Conclusions

Considering efficacy, safety, and cost-effectivenes, linezolid and tedizolid showed their superiority in MRSA cSSSI; while linezolid might be recommended to treat MRSA pneumonia. Although vancomycin was not cost-effective in pharmacoeconomic evaluation, it is still the first-line treatment for MRSA infection in the clinical practice. The approach used in this study might assist in revisiting therapeutic choices for patients with MRSA infections while awaiting the arrival of higher quality evidence.

## Additional file

Additional file 1: Supplementary material of network meta-analysis and pharmacoeconomic evaluation. (DOCX 2848 kb)

## Abbreviations

ABSSSI: Acute bacterial skin and skin structure infections
CA-MRSA: Community-associated MRSA
CE: Clinically evaluable
CI: Confidence interval
cSSSI: Complicated skin and soft structure infections
FDA: Food and Drug Administration
HAP: Hospital-acquired pneumonia
ICER: Incremental cost-effectiveness ratio
IDSA: The Infectious Diseases Society of America
ITT: Intention-to-treat
LYs: Life years
ME: Microbiologically evaluable
MIC: Minimum inhibitory concentration
mITT: Modified intention-to-treat
MRSA: Methicillin-resistant *Staphylococcus aureus*
NMA: Network meta-analysis
OR: Odds ratio
PRISMA: Preferred reporting items for systematic reviews and meta-analyses
PSA: Probabilistic sensitivity analysis
Q/D: Quinupristin/dalfopristin
RCT: Randomized controlled trial
SUCRA: Surface under the cumulative ranking curve
SXT: Trimethoprim/sulfamethoxazole
TOC: Test of cure
WTP: Willingness-to-pay
