# Supplementary material for: Network meta-analysis and pharmacoeconomic evaluation of antibiotics for the treatment of patients infected with complicated skin and soft structure infection and hospital-acquired or ventilator-associated penumonia
Source: Antimicrob Resist Infect Control. 2019 May 6;8:72. doi: 10.1186/s13756-019-0518-2 (PMC6501412; doi:10.1186/s13756-019-0518-2)
Supplement: Supplementary file 1 — Supplementary material of network meta-analysis and pharmacoeconomic evaluation. (DOCX 2848 kb) [file 13756_2019_518_MOESM1_ESM.docx]

Supplementary Content

**Appendix A** Search strategy of electronic databases

**Appendix B** Details of the statistical analysis of cost-effectiveness analysis

**Appendix C** Details of the statistical analysis of meta-analysis

**Appendix D** Trials characteristics in the network meta-analysis

**Appendix E** Risk of bias of included studies

**Appendix** **F** Results of meta-analysis for MRSA cSSSI

**Appendix G** Results of meta-analysis for MRSA pneumonia

**Appendix H** Assessment of loop inconsistency in networks

**Appendix I** Comparison-adjusted funnel plots for MRSA infections

**Appendix A**

Table A.1 Search strategies of electronic databases (PubMed as sample).

| **Database** |  | **Search strategy** |
| --- | --- | --- |
| PubMed | #1 | vancomycin [MeSH Terms] |
|  | #2 | linezolid [MeSH Terms] |
|  | #3 | teicoplanin [MeSH Terms] |
|  | #4 | daptomycin [MeSH Terms] |
|  | #5 | telavancin [Supplementary Concept] |
|  | #6 | dalbavancin [Supplementary Concept] |
|  | #7 | oritavancin [Supplementary Concept] |
|  | #8 | tigecycline [Supplementary Concept] |
|  | #9 | tedizolid phosphate [Supplementary Concept] |
|  | #10 | T91825 [Supplementary Concept] |
|  | #11 | JNJ-Q2 [Supplementary Concept] |
|  | #12 | #1 OR #2 OR #3 OR #4 OR #5 OR #6 OR #7 OR #8 OR #9 OR #10 OR #11 |
|  | #13 | Methicillin-resistant Staphylococcus aureus [MeSH Terms] |
|  | #14 | gram-positive bacterial infections [MeSH Terms] |
|  | #15 | pneumonia [Text Word] |
|  | #16 | skin and soft tissue infections [Text Word] |
|  | #17 | #13 OR #14 OR #15 OR #16 |
|  | #18 | random* [Text Word] |
|  | #19 | Randomized Controlled Trial [Publication Type] |
|  | #20 | Randomized Controlled trials as topic [MeSH Terms] |
|  | #21 | Controlled Clinical Trial [Publication Type] |
|  | #22 | randomized [Title/Abstract] |
|  | #23 | randomly [Title/Abstract] |
|  | #24 | groups [Title/Abstract] |
|  | #25 | #18 OR #19 OR #20 OR #21 OR #22 OR #23 #24 |
|  | #26 | #12 AND #17 AND #25 |

**Appendix B**

Text B.1 Details of the statistical analysis of cost-effectiveness analysis

Cost-effectiveness analysis

*Model structure*

Using TreeAge Pro 2011 (TreeAge Software, Inc., MA, USA), we conducted a decision-analytic model to assess the subsequent outcomes of antibiotic therapy with in patients with MRSA infections. The model was developed from the patient perspective in the United States. The decision tree model followed first-line and second-line therapy for MRSA cSSSI or HAP/VAP in Figure S2.

Ten therapeutic agents were included in the MRSA cSSSI model. The NMA showed that fusidic acid and teicoplanin were less effective; JNJ-Q2 was not marketed; although omadacycline was approved by FDA, it was not marketed currently; and sale of ceftobiprole has been discontinued, therefore they were excluded. Vancomycin, telavancin, dalbavancin, oritavancin, ceftaroline, tedizolid, tigecycline, daptomycin, trimethoprim/sulfamethoxazole plus rifampicin (SXT/rifampicin) were included in the cSSSI model. If the therapeutic efficacy of these drugs was not enough, we switched to linezolid. if linezolid was used as a treatment agent with poor efficacy, we switched to tedizolid. We used this strategy as the efficacy of linezolid and tedizolid were relatively large in the NMA.

Six therapeutic agents were included in the MRSA pneumonia model. According to the NMA, vancomycin plus rifampicin had poor clinical response; and sale of ceftobiprole has been discontinued, therefore these drugs were excluded. Vancomycin, linezolid, telavancin, Q/D, SXT/rifampin were evaluated in this model. If the efficacy of these drugs was insufficient, we switched to teicoplanin. If teicoplanin treatment was associated with poor efficacy, we switched to linezolid. We used this strategy as the efficacy of teicoplanin and linezolid was superior in the treatment of pneumonia.

*Model inputs*

Vancomycin was considered as comparator in the MRSA cSSSI model and the MRSA pneumonia model. The cure rate of the first-line treatment for the comparator was extracted from the pooled data of a pairwise meta-analysis. The ORs of clinical cure for the comparator versus each antibiotic were obtained from NMA to generate the probabilities for comparator agents. We calculated the probabilities of treatment switch using the clinical cure rates. Patients initiated on first-line treatment who required a therapy change to second-line treatment after 7 days of therapy in our base case. Other key parameters included in the model were length of hospital stay, inpatient and outpatient resource use and associated costs (i.e., inpatient laboratory cost, therapeutic drug monitoring (TDM) cost and physician outpatient cost), drug costs, incidence of serious adverse events (i.e., nephrotoxicity and thrombocytopenia)[1-5, 7, 10-27]. All costs are presented in 2017 US dollars with a conversion rate of 3%[28].

*Outcomes and Sensitivity analysis*

The key outcomes of interest were lifetime costs and life-years (LYs) saved for the two types of MRSA infections. The incremental cost-effectiveness ratio (ICER) per additional LY saved was calculated, which was used to compare the performance of treatment strategies. We did not adjust for quality of life because we assumed that survivors of MRSA infections are unlikely to have long-term consequences related to this condition (i.e., utility value=1)[29]. We considered each treatment strategy resulting in an effect size less than the willingness-to-pay (WTP) threshold ($50,000) to be acceptable[30].

Deterministic sensitivity analyses were conducted to assess the influence of fluctuation of key parameters on the ICERs. Probabilistic sensitivity analyses (PSA) were also performed to evaluate all uncertainties about model inputs simultaneously using 1,000 second-order Monte Carlo simulations.

Figure B.1 Decision tree model.


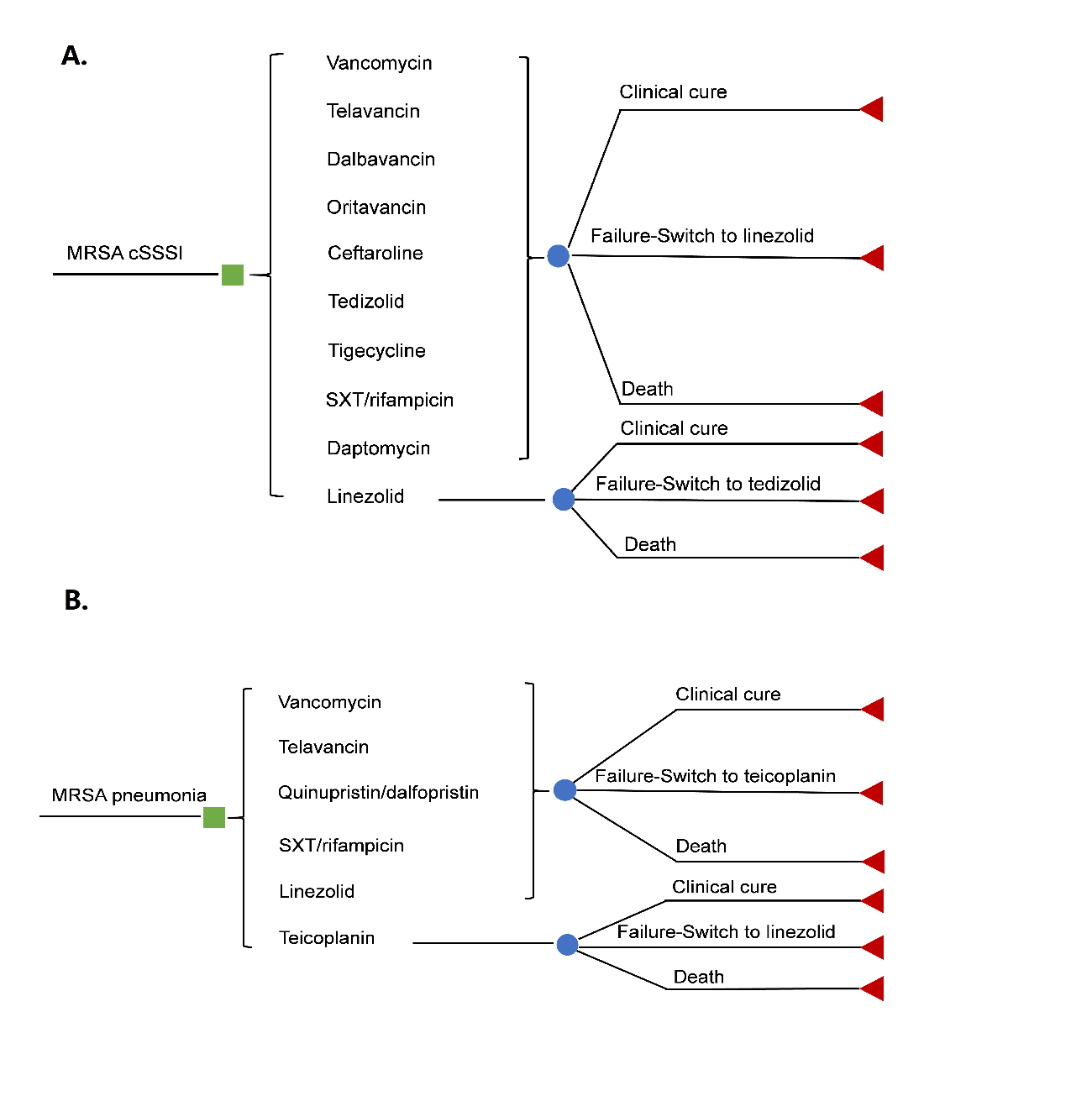


MRSA, methicillin-resistant Staphylococcus aureus. cSSSI, complicated skin and soft structure infection. SXT, trimethoprim/sulfamethoxazole.

A. Decision tree model for MRSA cSSSI. B. Decision tree model for MRSA pneumonia

Table B.1 Model input parameters

*Model input parameters for MRSA cSSSI*

| Parameter | Base case | Sensitivity Range | References |
| --- | --- | --- | --- |
| Model inputs |  |  |  |
| Cure rate |  |  |  |
| Tedizolid | 0.83 | 0.40-0.92 (Beta) | Pooled data |
| OR of cure for tedizolid versus antibiotics |  |  |  |
| Linezolid | 0.90 | 0.55-1.23 (Lognormal) | NMA |
| Vancomycin | 1.39 | 0.63-2.12 (Lognormal) | NMA |
| Telavancin | 1.25 | 0.48-1.56 (Lognormal) | NMA |
| Tigecycline | 1.65 | 0.49-2.17 (Lognormal) | NMA |
| SXT/rifampicin | 0.57 | 0.48-1.90 (Lognormal) | NMA |
| Daptomycin | 1.46 | 0.79-2.17 (Lognormal) | NMA |
| Oritavancin | 1.15 | 0.53-2.13 (Lognormal) | NMA |
| Dalbavancin | 1.31 | 0.42-2.57 (Lognormal) | NMA |
| Ceftaroline | 1.31 | 0.64-2.65 (Lognormal) | NMA |
| Mortality |  |  |  |
| Tedizolid | 0.004 | 0.001-0.005 (Beta) | NMA |
| Linezolid | 0.005 | 0.002-0.008 (Beta) | NMA |
| Vancomycin | 0.004 | 0.002-0.008 (Beta) | NMA |
| Telavancin | 0.004 | 0.002-0.007 (Beta) | NMA |
| Tigecycline | 0.006 | 0.003-0.009 (Beta) | NMA |
| SXT/rifampicin | 0.003 | 0.001-0.005 (Beta) | NMA |
| Daptomycin | 0.003 | 0.001-0.005 (Beta) | NMA |
| Oritavancin | 0.002 | 0.001-0.004 (Beta) | NMA |
| Dalbavancin | 0.002 | 0.001-0.004 (Beta) | NMA |
| Ceftaroline | 0.008 | 0.004-0.011 (Beta) | NMA |

| First-line treatment, failure |  |  | Calculation |
| --- | --- | --- | --- |
| Tedizolid | 0.16 |  |  |
| Linezolid | 0.13 |  |  |
| Vancomycin | 0.24 |  |  |
| Telavancin | 0.22 |  |  |
| Tigecycline | 0.26 |  |  |
| SXT/rifampicin | 0.02 |  |  |
| Daptomycin | 0.25 |  |  |
| Oritavancin | 0.19 |  |  |
| Dalbavancin | 0.22 |  |  |
| Ceftaroline | 0.23 |  |  |
| AEs requiring therapy switch |  |  |  |
| Nephrotoxicity |  |  |  |
| Tedizolid | NA |  |  |
| Linezolid | 0.08 | 0.024-0.091 (Beta) | Supplementary material [39] |
| Vancomycin | 0.18 | 0.122-0.201 (Beta) | Supplementary material [39] |
| Telavancin | 0.10 | 0.082-0.183 (Beta) | Supplementary material [23] |
| Tigecycline | NA |  |  |
| SXT/rifampicin | 0.02 | 0.014-0.031 (Beta) | Supplementary material [3] |
| Daptomycin | 0.03 | 0.016-0.043 (Beta) | Supplementary material [51] |
| Oritavancin | NA |  |  |
| Dalbavancin | NA |  |  |
| Ceftaroline | NA |  |  |
| Thrombocytopenia |  |  |  |
| Tedizolid | NA |  |  |
| Linezolid | 0.16 | 0.123-0.226 (Beta) | Supplementary material [39] |
| Vancomycin | 0.13 | 0.091-0.183 (Beta) | Supplementary material [39] |
| Telavancin | NA |  |  |
| Tigecycline | NA |  |  |
| SXT/rifampicin | NA |  |  |
| Daptomycin | NA |  |  |
| Oritavancin | NA |  |  |
| Dalbavancin | NA |  |  |
| Ceftaroline | NA |  |  |
| Nausea |  |  |  |
| Tedizolid | 0.09 | 0.048-0.132 (Beta) | Supplementary material [22] |
| Linezolid | 0.13 | 0.092-0.173 (Beta) | Supplementary material [22] |
| Vancomycin | 0.09 | 0.051-0.122 (Beta) | Supplementary material [5] |
| Telavancin | 0.14 | 0.082-0.175 (Beta) | Supplementary material [23] |
| Tigecycline | 0.25 | 0.221-0.323 (Beta) | Supplementary material [24] |
| SXT/rifampicin | 0.13 | 0.082-0.163 (Beta) | Supplementary material [3] |
| Daptomycin | 0.06 | 0.022-0.081 (Beta) | Supplementary material [51] |
| Oritavancin | 0.11 | 0.075-0.163 (Beta) | Supplementary material [5] |
| Dalbavancin | 0.03 | 0.018-0.072 (Beta) | Supplementary material [6] |
| Ceftaroline | 0.04 | 0.018-0.082 (Beta) | Supplementary material [27] |
| Diarrhoea |  |  |  |
| Tedizolid | 0.05 | 0.025-0.078 (Beta) | Supplementary material [22] |
| Linezolid | 0.05 | 0.024-0.081 (Beta) | Supplementary material [22] |
| Vancomycin | 0.09 | 0.032-0.125 (Beta) | Supplementary material [5] |
| Telavancin | 0.08 | 0.027-0.105 (Beta) | Supplementary material [23] |
| Tigecycline | 0.04 | 0.019-0.085 (Beta) | Supplementary material [24] |
| SXT/rifampicin | 0.05 | 0.018-0.081 (Beta) | Supplementary material [3] |
| Daptomycin | 0.02 | 0.009-0.042 (Beta) | Supplementary material [25] |
| Oritavancin | 0.05 | 0.024-0.083 (Beta) | Supplementary material [5] |
| Dalbavancin | 0.01 | 0.008-0.035 (Beta) | Supplementary material [6] |
| Ceftaroline | 0.02 | 0.009-0.048 (Beta) | Supplementary material [27] |
|  |  |  |  |
| Cost (USD) |  |  |  |
| Tedizolid (200mg IV) | 400 | 292-725 (Gamma) |  |
| Vancomycin (1g IV) | 22 | 9-52 (Gamma) | Supplementary material [21] |
| Linezolid (600mg IV) | 157 | 89-221 (Gamma) | Supplementary material [21] |
| Telavancin (750mg IV) | 467 | 278-680 (Gamma) | Supplementary material [21] |
| Oritavancin (500mg IV) | 1987 | 1020-3567 (Gamma) | Supplementary material [21] |
| Dalbavancin (500mg IV) | 1841 | 988-3024 (Gamma) | Supplementary material [21] |
| Daptomycin (500mg IV) | 250 | 163-479 (Gamma) | Supplementary material [21] |
| Tigecycline (50mg IV) | 67 | 34-98 (Gamma) | Supplementary material [21] |
| SXT/rifampicin (SXT:160mg/800mg rifampicin:300mg IV) | 2.87 | 1.95-6.78 (Gamma) | Supplementary material [21] |
| Ceftaroline (600mg IV) | 171 | 85-245 (Gamma) | Supplementary material [21] |
| Daily hospitalization costs | 1233 | 888-2934 (Gamma) | Supplementary material [22] |
| TDM | 51 | 34-98 (Gamma) | Supplementary material [23] |
| Laboratory test | 100 | 67-179 (Gamma) | Supplementary material [24] |
| Physician outpatient visit | 204 | 136-474 (Gamma) | Supplementary material [25] |
| AEs cost |  |  |  |
| Nephrotoxicity | 3052 | 1500-4552 (Gamma) | Supplementary material [20] |
| Thrombocytopenia | 914 | 450-1360 (Gamma) | Supplementary material [21] |
| Diarrhea | 702 | 340-1002 (Gamma) | Supplementary material [62] |
| Nausea | 22 | 10-35 (Gamma) | Supplementary material [63] |
|  |  |  |  |
| Other parameters |  |  |  |
| Hospital stay, days |  |  |  |
| Tedizolid | 6 | 5-10 (Triangular) | Supplementary material [7] |
| Linezolid | 7 | 5-14 (Triangular) | Supplementary material [8] |
| Vancomycin | 8 | 7-14 (Triangular) | Supplementary material [16] |
| Telavancin | 8 | 7-14 (Triangular) | Supplementary material [16] |
| Tigecycline | 8 | 6-14 (Triangular) | Supplementary material [8] |
| SXT/rifampicin | 10 | 7-14 (Triangular) | Supplementary material [9] |
| Daptomycin | 6 | 4-10 (Triangular) | Supplementary material [10] |
| Oritavancin | 6 | 5-10 (Triangular) | Supplementary material [11] |
| Dalbavancin | 8 | 7-14 (Triangular) | Supplementary material [12] |
| Ceftaroline | 7 | 5-14 (Triangular) | Supplementary material [13] |
| Treatment days before discontinuing first-line treatment |  |  | Physician survey |
| Tedizolid | 7 |  |  |
| Linezolid | 7 |  |  |
| Vancomycin | 7 |  |  |
| Telavancin | 7 |  |  |
| Tigecycline | 7 |  |  |
| SXT/rifampicin | 7 |  |  |
| Daptomycin | 7 |  |  |
| Oritavancin | 7 |  |  |
| Dalbavancin | 7 |  |  |
| Ceftaroline | 7 |  |  |
| LYs |  |  |  |
| cSSSI | 8 |  | Supplementary material [60] |

MRSA, methicillin-resistant Staphylococcus aureus. cSSSI, complicated skin and soft structure infection. OR, odds ratio. NMA, network meta-analysis. SXT, trimethoprim/sulfamethoxazole. AEs, adverse events. NA, not available. USD, US dollar. IV, intravenous. TDM, therapeutic drug monitoring. LYs, life-years.

*Model input parameters for MRSA pneumonia*

| Parameter | | Base case | | Sensitivity Range | | References | |
| --- | --- | --- | --- | --- | --- | --- | --- |
| Model inputs | | | | | | | |
| First-line treatment, success | | | | | | | |
| Cure rate | | | | | | | |
| SXT/rifampicin | | 0.55 | | 0.29-0.70 (Beta) | | Pooled data | |
| OR of cure for SXT/rifampicin versus antibiotics | | | | | | | |
| Linezolid | | 0.42 | | 0.05-2.31 (Lognormal) | | NMA | |
| Vancomycin | | 0.50 | | 0.06-1.44 (Lognormal) | | NMA | |
| Telavancin | | 0.52 | | 0.16-1.32 (Lognormal) | | NMA | |
| Quinupristin/dalfopristin | | 0.54 | | 0.18-2.01 (Lognormal) | | NMA | |
| Teicoplanin | | 0.31 | | 0.09-2.56 (Lognormal) | | NMA | |
| Mortality | |  | |  | |  | |
| SXT/rifampicin | | 0.04 | | 0.013-0.052 (Beta) | | NMA | |
| Linezolid | | 0.07 | | 0.044-0.092 (Beta) | | NMA | |
| Vancomycin | | 0.18 | | 0.143-0.212 (Beta) | | NMA | |
| Telavancin | | 0.20 | | 0.185-0.233 (Beta) | | NMA | |
| Quinupristin/dalfopristin | | 0.15 | | 0.131-0.182 (Beta) | | NMA | |
| Teicoplanin | | 0.22 | | 0.167-0.254 (Beta) | | NMA | |
| First-line treatment, failure | |  | |  | | Calculation | |
| SXT/rifampicin | | 0.40 | |  | |  | |
| Linezolid | | 0.23 | |  | |  | |
| Vancomycin | | 0.22 | |  | |  | |
| Telavancin | | 0.11 | |  | |  | |
| Quinupristin/dalfopristin | | 0.25 | |  | |  | |
| Teicoplanin | | 0.47 | |  | |  | |
| AEs requiring therapy switch | |  | |  | |  | |
| Nephrotoxicity | |  | |  | |  | |
| SXT/rifampicin | | NA | |  | |  | |
| Linezolid | | 0.08 | | 0.035-0.121 (Beta) | | Supplementary material [11] | |
| Vancomycin | | 0.18 | | 0.132-0.230 (Beta) | | Supplementary material [11] | |
| Telavancin | | 0.10 | | 0.083-0.147 (Beta) | | Supplementary material [12] | |
| Quinupristin/dalfopristin | | NA | |  | |  | |
| Teicoplanin | | 0.05 | | 0.019-0.055 (Beta) | | Supplementary material [55] | |
| Thrombocytopenia | |  | |  | |  | |
| SXT/rifampicin | | NA | |  | |  | |
| Linezolid | | 0.16 | | 0.132-0.189 (Beta) | | Supplementary material [11] | |
| Vancomycin | | 0.13 | | 0.102-0.157 (Beta) | | Supplementary material [11] | |
| Telavancin | | NA | |  | |  | |
| Quinupristin/dalfopristin | | NA | |  | |  | |
| Teicoplanin | | NA | |  | |  | |
| Nausea | |  | |  | |  | |
| SXT/rifampicin | | 0.13 | | 0.097-0.178 (Beta) | | Supplementary material [3] | |
| Linezolid | | 0.03 | | 0.013-0.065 (Beta) | | Supplementary material [40] | |
| Vancomycin | | 0.04 | | 0.012-0.087 (Beta) | | Supplementary material [12] | |
| Telavancin | | 0.05 | | 0.102-0.157 (Beta) | | Supplementary material [12] | |
| Quinupristin/dalfopristin | | NA | |  | |  | |
| Teicoplanin | | NA | |  | |  | |
| Diarrhoea | |  | |  | |  | |
| SXT/rifampicin | | 0.05 | | 0.013-0.085 (Beta) | | Supplementary material [3] | |
| Linezolid | | 0.01 | | 0.079-0.043 (Beta) | | Supplementary material [40] | |
| Vancomycin | | 0.12 | | 0.102-0.148 (Beta) | | Supplementary material [12] | |
| Telavancin | | 0.11 | | 0.089-0.137 (Beta) | | Supplementary material [12] | |
| Quinupristin/dalfopristin | | NA | |  | |  | |
| Teicoplanin | | 0.04 | | 0.013-0.077 (Beta) | | Supplementary material [55] | |
| Cost (USD) | |  | |  | |  | |
| Vancomycin (1g IV) | | 22 | | 9-52 (Gamma) | | Supplementary material [21] | |
| Linezolid (600mg IV) | | 157 | | 89-221 (Gamma) | | Supplementary material [21] | |
| Telavancin (750mg IV) | | 467 | | 278-680 (Gamma) | | Supplementary material [21] | |
| SXT/rifampicin (SXT:160mg/800mg rifampicin:300mg IV) | | 2.87 | | 1.95-6.78 (Gamma) | | Supplementary material [21] | |
| Quinupristin/dalfopristin (500mg IV) | | 284 | | 178-400 (Gamma) | | Supplementary material [21] | |
| Teicoplanin (400mg IV) | | 60 | | 29-96 (Gamma) | | Supplementary material [21] | |
| Daily hospitalization costs | | 1233 | | 888-2934 (Gamma) | | Supplementary material [22] | |
| TDM | | 51 | | 34-98 (Gamma) | | Supplementary material [23] | |
| Laboratory test | | 100 | | 67-179 (Gamma) | | Supplementary material [24] | |
| Physician outpatient visit | | 204 | | 136-474 (Gamma) | | Supplementary material [25] | |
| AEs cost | |  | |  | |  | |
| Nephrotoxicity | | 3052 | |  | | Supplementary material [20] | |
| Thrombocytopenia | | 914 | |  | | Supplementary material [21] | |
| Diarrhea | | 702 | |  | | Supplementary material [62] | |
| Nausea | | 22 | |  | | Supplementary material [63] | |
|  | |  | |  | |  | |
| Other parameters | |  | |  | |  | |
| Hospital stay, days | |  | |  | |  | |
| SXT/rifampicin | | 10 | | 7-14 (Triangular) | | Supplementary material [9] | |
| Linezolid | | 10 | | 7-14 (Triangular) | | Supplementary material [20] | |
| Vancomycin | | 10 | | 7-14 (Triangular) | | Supplementary material [20] | |
| Telavancin | | 10 | | 7-14 (Triangular) | | Supplementary material [18] | |
| Quinupristin/dalfopristin | | 11 | | 7-14 (Triangular) | | Supplementary material [15] | |
| Teicoplanin | | 8 | | 7-14 (Triangular) | | Supplementary material [8] | |
| Treatment days before discontinuing first-line treatment | |  | |  | | Physician survey | |
| SXT/rifampicin | | 7 | |  | |  | |
| Linezolid | | 7 | |  | |  | |
| Vancomycin | | 7 | |  | |  | |
| Telavancin | | 7 | |  | |  | |
| Quinupristin/dalfopristin | | 7 | |  | |  | |
| Teicopalnin | | 7 | |  | |  | |
| LYs | |  | |  | |  | |
| HAP/VAP | | 10 | |  | | Supplementary material [61] | |

MRSA, methicillin-resistant Staphylococcus aureus. SXT, trimethoprim/sulfamethoxazole. OR, odds ratio. NMA, network meta-analysis. AE, adverse events. NA, not available. USD, US dollar. IV, intravenous. TDM, therapeutic drug monitoring. LYs, life-years. HAP/VAP, hospital-acquired or ventilator-associated pneumonia.

Ap**pendix C**

**Text C.1** Details of the statistical analysis of meta-analysis

We conducted two types of meta-analyses. First, we performed pairwise meta-analysis with a random-effects model[31]. We report the estimates of primary and secondary outcomes using odds ratio (OR) and their corresponding 95% confidence intervals (CI). P <0.05 was considered to be statistically significant. We assessed statistical heterogeneity across trials with the *I^2^* statistic[32]. *I^2^* >50% indicated significant heterogeneity. Secondly, we conducted a random-effects network meta-analysis for direct and indirect comparisons[33, 34]. We estimated the effects in our meta-analyses by using odds ratio (OR) and 95% confidence intervals (CI). A common estimate (the tau [τ] value) was assumed to assess the heterogeneity among all comparisons. We investigated the degree of heterogeneity through comparing the magnitude of τ[35]. In order to estimate the treatment rank for all interventions, the surface under the cumulative ranking curve probabilities was reported for all outcomes[36]. Transitivity is a basic assumption in a network meta-analysis. Transitivity means that we can learn about treatment A versus B via a common comparator C. If our network contained a closed loop, we utilized the transitivity assumption to assess consistency by comparing direct and indirect overall effects.

To examine the discrepancy between trials affected by study characteristics, we conducted a sensitivity network meta-analyses for primary outcomes according to the following variables: sex ratio, treatment duration, number of RCTs. We used Stata (Version 13.0) for the analyses.

References

[1] Prokocimer P, De Anda C, Fang E, Mehra P, Das A. 2013. Tedizolid phosphate vs linezolid for treatment of acute bacterial skin and skin structure infections: the ESTABLISH-1 randomized trial. JAMA 309: 559-569.

[2] Florescu I, Beuran M, Dimov R, Razbadauskas A, Bochan M, Fichev G, Dukart G, Babinchak T, Cooper CA, Ellis-Grosse EJ, Dartois N, Gandjini H. 2008. Efficacy and safety of tigecycline compared with vancomycin or linezolid for treatment of serious infections with methicillin-resistant Staphylococcus aureus or vancomycin-resistant enterococci: a Phase 3, multicentre, double-blind, randomized study. J Antimicrob Chemother 62 Suppl 1: i17-28.

[3] Harbarth S, von Dach E, Pagani L, Macedo-Vinas M, Huttner B, Olearo F, Emonet S, Uçkay I. 2015. Randomized non-inferiority trial to compare trimethoprim/sulfamethoxazole plus rifampicin versus linezolid for the treatment of MRSA infection. J Antimicrob Chemother 70: 264-272.

[4] Pertel PE, Eisenstein BI, Link AS, Donfrid B, Biermann EJ, Bernardo P, Martone WJ. 2009. The efficacy and safety of daptomycin vs. vancomycin for the treatment of cellulitis and erysipelas. International Journal of Clinical Practice 63: 368-375.

[5] Corey GR, Kabler H, Mehra P, Gupta S, Overcash JS, Porwal A, Giordano P, Lucasti C, Perez A, Good S, Jiang H, Moeck G, O'Riordan W. 2014. Single-dose oritavancin in the treatment of acute bacterial skin infections. N Engl J Med 370: 2180-2190.

[6] Boucher HW, Wilcox M, Talbot GH, Puttagunta S, Das AF, Dunne MW. 2014. Once-weekly dalbavancin versus daily conventional therapy for skin infection pooled study (DISCOVERY 1 and 2). N Engl J Med 370: 2169-2179.

[7] Wilcox MH, Corey GR, Talbot GH, Thye D, Friedland D, Baculik T. 2010. CANVAS 2: the second Phase III, randomized, double-blind study evaluating ceftaroline fosamil for the treatment of patients with complicated skin and skin structure infections. J Antimicrob Chemother 65 Suppl 4: iv53-65.

[8] Wilcox M, Nathwani D, Dryden M. 2004. Linezolid compared with teicoplanin for the treatment of suspected or proven Gram-positive infections. J Antimicrob Chemother 53: 335-344.

[9] Fagon J, Patrick H, Haas DW, Torres A, Gibert C, Cheadle WG, Falcone RE, Anholm JD, Paganin F, Fabian TC, Lilienthal F. 2000. Treatment of gram-positive nosocomial pneumonia. Prospective randomized comparison of quinupristin/dalfopristin versus vancomycin. Nosocomial Pneumonia Group. Am J Respir Crit Care Med 161: 753-762.

[10] Stryjewski ME, Graham DR, Wilson SE, O'Riordan W, Young D, Lentnek A, Ross DP, Fowler VG, Hopkins A, Friedland HD, Barriere SL, Kitt MM, Corey GR. 2008. Telavancin versus vancomycin for the treatment of complicated skin and skin-structure infections caused by gram-positive organisms. Clin Infect Dis 46: 1683-1693.

[11] Wunderink RG, Niederman MS, Kollef MH, Shorr AF, Kunkel MJ, Baruch A, McGee WT, Reisman A, Chastre J. 2012. Linezolid in methicillin-resistant Staphylococcus aureus nosocomial pneumonia: a randomized, controlled study. Clin Infect Dis 54: 621-629.

[12] Rubinstein E, Lalani T, Corey GR, Kanafani ZA, Nannini EC, Rocha MG, Rahav G, Niederman MS, Kollef MH, Shorr AF, Lee PC, Lentnek AL, Luna CM, Fagon JY, Torres A, Kitt MM, Genter FC, Barriere SL, Friedland HD, Stryjewski ME. 2011. Telavancin versus vancomycin for hospital-acquired pneumonia due to gram-positive pathogens. Clin Infect Dis 52: 31-40.

[13] Katz DE, Lindfield KC, Steenbergen JN, Benziger DP, Blackerby KJ, Knapp AG, Martone WJ. 2008. A pilot study of high-dose short duration daptomycin for the treatment of patients with complicated skin and skin structure infections caused by gram-positive bacteria. Int J Clin Pract 62: 1455-1464.

[14] Lin DF, Zhang YY, Wu JF, Wang F, Zheng JC, Miao JZ, Zheng LY, Sheng RY, Zhou X, Shen HH, Ijzerman MM, Croos-Dabrera RV, Sheng W. 2008. Linezolid for the treatment of infections caused by Gram-positive pathogens in China. Int J Antimicrob Agents 32: 241-249.

[15] USA Registered Pharmacy [online] 2017 Available from: https://rxusa.com.

[16] Milbrandt EB, Kersten A, Rahim MT, Dremsizov TT, Clermont G, Cooper LM, Angus DC, Linde-Zwirble WT. 2008. Growth of intensive care unit resource use and its estimated cost in Medicare. Crit Care Med 36: 2504-2510.

[17] Ingenix. National Fee Analyzer. Eden Prairie, MN: Ingenix; 2011

[18] Shillcutt SD1, Walker DG, Goodman CA, Mills AJ. 2010. Cost-effectiveness in low- and middle-income countries. Pharmacoeconomics 27: 903-917.

[19] Tice AD, Hoaglund PA, Nolet B, McKinnon PS, Mozaffari E. 2002. Cost perspectives for outpatient intravenous antimicrobial therapy. Pharmacotherapy 22: 63-70.

[20] Chertow GM1, Burdick E, Honour M, Bonventre JV, Bates DW. 2005. Acute kidney injury, mortality, length of stay, and costs in hospitalized patients. J Am Soc Nephrol 16: 3365-3370.

[21] Smythe MA, Koerber JM, Fitzgerald M, Mattson JC. 2008. The financial impact of heparin-induced thrombocytopenia. Chest 134: 568-573.

[22] Moran GJ, Fang E, Corey GR, Das AF, De Anda C, Prokocimer P. 2014. Tedizolid for 6 days versus linezolid for 10 days for acute bacterial skin and skin-structure infections (ESTABLISH-2): a randomised, double-blind, phase 3, non-inferiority trial. Lancet Infect Dis 14: 696-705.

[23] Stryjewski ME, Potgieter PD, Li YP, Barriere SL, Churukian A, Kingsley J, Corey GR. 2012. TD-1792 versus vancomycin for treatment of complicated skin and skin structure infections. Antimicrob Agents Chemother 56: 5476-5483.

[24] Breedt J, Teras J, Gardovskis J, Maritz FJ, Vaasna T, Ross DP, Gioud-Paquet M, Dartois N, Ellis-Grosse EJ, Loh E. 2005. Safety and efficacy of tigecycline in treatment of skin and skin structure infections: results of a double-blind phase 3 comparison study with vancomycin-aztreonam. Antimicrob Agents Chemother 49: 4658-4666.

[25] Aikawa N, Kusachi S, Mikamo H, Takesue Y, Watanabe S, Tanaka Y, Morita A, Tsumori K, Kato Y, Yoshinari T. 2013. Efficacy and safety of intravenous daptomycin in Japanese patients with skin and soft tissue infections. J Infect Chemother 19: 447-455.

[26] Corey GR, Good S, Jiang H, Moeck G, Wikler M, Green S, Manos P, Keech R, Singh R, Heller B, Bubnova N, O'Riordan W. 2015. Single-dose oritavancin versus 7-10 days of vancomycin in the treatment of gram-positive acute bacterial skin and skin structure infections: The SOLO II noninferiority study. Clin Infect Dis 60: 254-262.

[27] Dryden M, Zhang Y, Wilson D, Iaconis JP, Gonzalez J. 2016. A Phase III, randomized, controlled, non-inferiority trial of ceftaroline fosamil 600 mg every 8 h versus vancomycin plus aztreonam in patients with complicated skin and soft tissue infection with systemic inflammatory response or underlying comorbidities. J Antimicrob Chemother 71: 3575-3584.

[28] Gold JES MR, Russell LB, Weinstein MC. New York: Oxford University Press; 1996

[29] von Dach E, Morel CM, Murthy A, Pagani L, Macedo-Vinas M, Olearo F, Harbarth S. 2017. Comparing the cost-effectiveness of linezolid to trimethoprim/sulfamethoxazole plus rifampicin for the treatment of methicillin resistant Staphylococcus aureus infection: a healthcare system perspective. Clin Microbiol Infect 23: 659-666.

[30] Birch S, Gafni A. 2006. Information created to evade reality (ICER): things we should not look to for answers. Pharmacoeconomics 24:1121-1131.

[31] DerSimonian R, Laird N. 1986. Meta-analysis in clinical trials. Control Clin Trials 7: 177-188.

[32] Higgins JP, Thompson SG, Deeks JJ, Altman DG. 2003. Measuring inconsistency in meta-analyses. BMJ 327: 557-560.

[33] Cipriani A, Higgins JP, Geddes JR, Salanti G. 2013. Conceptual and technical challenges in network meta-analysis. Ann Intern Med 159: 130-137.

[34] Caldwell DM, Ades AE, Higgins JP. 2005. Simultaneous comparison of multiple treatments: combining direct and indirect evidence. BMJ 331: 897-900.

[35] Turner RM, Davey J, Clarke MJ, Thompson SG, Higgins JP. 2012. Predicting the extent of heterogeneity in meta-analysis, using empirical data from the Cochrane Database of Systematic Reviews. Int J Epidemiol 41: 818-827.

[36] Chaimani A, Higgins JP, Mavridis D, Spyridonos P, Salanti G. 2013. Graphical tools for network meta-analysis in STATA. PLoS One 8: e76654.

[37] Itani KM, Dryden MS, Bhattacharyya H, Kunkel MJ, Baruch AM, Weigelt JA. 2010. Efficacy and safety of linezolid versus vancomycin for the treatment of complicated skin and soft-tissue infections proven to be caused by methicillin-resistant Staphylococcus aureus. Am J Surg 199: 804-816.

[38] Wunderink RG, Mendelson MH, Somero MS, Fabian TC, May AK, Bhattacharyya H, Leeper KV Jr, Solomkin JS. 2008. Early microbiological response to linezolid vs vancomycin in ventilator-associated pneumonia due to methicillin-resistant Staphylococcus aureus. Chest 134: 1200-1207.

[39] Kohno S, Yamaguchi K, Aikawa N, Sumiyama Y, Odagiri S, Aoki N, Niki Y, Watanabe S, Furue M, Ito T, Croos-Dabrera R, Tack KJ. 2007. Linezolid versus vancomycin for the treatment of infections caused by methicillin-resistant Staphylococcus aureus in Japan. J Antimicrob Chemother 60: 1361-1369.

[40] Jaksic B, Martinelli G, Perez-Oteyza J, Hartman CS, Leonard LB, Tack KJ. 2006. Efficacy and safety of linezolid compared with vancomycin in a randomized, double-blind study of febrile neutropenic patients with cancer. Clin Infect Dis 42: 597-607.

[41] Weigelt J, Itani K, Stevens D, Lau W, Dryden M, Knirsch C. 2005. Linezolid versus vancomycin in treatment of complicated skin and soft tissue infections. Antimicrob Agents Chemother 49: 2260-2266.

[42] Wunderink RG, Cammarata SK, Oliphant TH, Kollef MH. 2003. Continuation of a randomized, double-blind, multicenter study of linezolid versus vancomycin in the treatment of patients with nosocomial pneumonia. Clin Ther 25: 980-992.

[43] Stevens DL, Herr D, Lampiris H, Hunt JL, Batts DH, Hafkin B. 2002. Linezolid versus vancomycin for the treatment of methicillin-resistant Staphylococcus aureus infections. Clin Infect Dis 34: 1481-1490.

[44] Rubinstein E, Cammarata S, Oliphant T, Wunderink R. 2001. Linezolid (PNU-100766) versus vancomycin in the treatment of hospitalized patients with nosocomial pneumonia: a randomized, double-blind, multicenter study. Clin Infect Dis 32: 402-412.

[45] Corey GR, Wilcox MH, Talbot GH, Thye D, Friedland D, Baculik T. 2010. CANVAS 1: the first Phase III, randomized, double-blind study evaluating ceftaroline fosamil for the treatment of patients with complicated skin and skin structure infections. J Antimicrob Chemother 65 Suppl 4: iv41-51.

[46] Talbot GH1, Thye D, Das A, Ge Y. 2007. Phase 2 study of ceftaroline versus standard therapy in treatment of complicated skin and skin structure infections. Antimicrob Agents Chemother 51: 3612-3616.

[47] Awad SS, Rodriguez AH, Chuang YC, Marjanek Z, Pareigis AJ, Reis G, Scheeren TW, Sánchez AS, Zhou X, Saulay M, Engelhardt M. 2014. A phase 3 randomized double-blind comparison of ceftobiprole medocaril versus ceftazidime plus linezolid for the treatment of hospital-acquired pneumonia. Clin Infect Dis 59: 51-61.

[48] Noel GJ, Strauss RS, Amsler K, Heep M, Pypstra R, Solomkin JS. 2008. Results of a double-blind, randomized trial of ceftobiprole treatment of complicated skin and skin structure infections caused by gram-positive bacteria. Antimicrob Agents Chemother 52: 37-44.

[49] Noel GJ, Bush K, Bagchi P, Ianus J, Strauss RS. 2008. A randomized, double-blind trial comparing ceftobiprole medocaril with vancomycin plus ceftazidime for the treatment of patients with complicated skin and skin-structure infections. Clin Infect Dis 46: 647-655.

[50] Jauregui LE, Babazadeh S, Seltzer E, Goldberg L, Krievins D, Frederick M, Krause D, Satilovs I, Endzinas Z, Breaux J, O'Riordan W. 2005. Randomized, double-blind comparison of once-weekly dalbavancin versus twice-daily linezolid therapy for the treatment of complicated skin and skin structure infections. Clin Infect Dis 41: 1407-1415.

[51] Arbeit RD, Maki D, Tally FP, Campanaro E, Eisenstein BI. 2004. The safety and efficacy of daptomycin for the treatment of complicated skin and skin-structure infections. Clin Infect Dis 38: 1673-1681.

[52] Stryjewski ME, Chu VH, O'Riordan WD, Warren BL, Dunbar LM, Young DM, Vallée M, Fowler VG Jr, Morganroth J, Barriere SL, Kitt MM, Corey GR. 2006. Telavancin versus standard therapy for treatment of complicated skin and skin structure infections caused by gram-positive bacteria: FAST 2 study. Antimicrob Agents Chemother 50: 862-867.

[53] Stryjewski ME, O'Riordan WD, Lau WK, Pien FD, Dunbar LM, Vallee M, Fowler VG Jr, Chu VH, Spencer E, Barriere SL, Kitt MM, Cabell CH, Corey GR. 2005. Telavancin versus standard therapy for treatment of complicated skin and soft-tissue infections due to gram-positive bacteria. Clin Infect Dis 40: 1601-1607.

[54] Sacchidanand S, Penn RL, Embil JM, Campos ME, Curcio D, Ellis-Grosse E, Loh E, Rose G. 2005. Efficacy and safety of tigecycline monotherapy compared with vancomycin plus aztreonam in patients with complicated skin and skin structure infections: Results from a phase 3, randomized, double-blind trial. Int J Infect Dis 9: 251-261.

[55] Cepeda JA, Whitehouse T, Cooper B, Hails J, Jones K, Kwaku F, Taylor L, Hayman S, Shaw S, Kibbler C, Shulman R, Singer M, Wilson AP. 2004. Linezolid versus teicoplanin in the treatment of Gram-positive infections in the critically ill: a randomized, double-blind, multicentre study. J Antimicrob Chemother 53: 345-355.

[56] Noel GJ, Draper MP, Hait H, Tanaka SK, Arbeit RD. 2012. A randomized, evaluator-blind, phase 2 study comparing the safety and efficacy of omadacycline to those of linezolid for treatment of complicated skin and skin structure infections. Antimicrob Agents Chemother 56: 5650-5654.

[57] Craft JC, Moriarty SR, Clark K, Scott D, Degenhardt TP, Still JG, Corey GR, Das A, Fernandes P. 2011. A randomized, double-blind phase 2 study comparing the efficacy and safety of an oral fusidic acid loading-dose regimen to oral linezolid for the treatment of acute bacterial skin and skin structure infections. Clin Infect Dis 52: S520-526.

[58] Covington P, Davenport JM, Andrae D, O'Riordan W, Liverman L, McIntyre G, Almenoff J. 2011. Randomized, double-blind, phase II, multicenter study evaluating the safety/tolerability and efficacy of JNJ-Q2, a novel fluoroquinolone, compared with linezolid for treatment of acute bacterial skin and skin structure infection. Antimicrob Agents Chemother 55: 5790-5797.

[59] Jung YJ, Koh Y, Hong SB, Chung JW, Ho Choi S, Kim NJ, Kim MN, Choi IS, Han SY, Kim WD, Yun SC, Lim CM. 2010. Effect of vancomycin plus rifampicin in the treatment of nosocomial methicillin-resistant Staphylococcus aureus pneumonia. Crit Care Med 38: 175-180.

[60] Hersh AL1, Chambers HF, Maselli JH, Gonzales R. 2008. National trends in ambulatory visits and antibiotic prescribing for skin and soft-tissue infections. Arch Intern Med 168: 1585-1591.

[61] Shorr AF, Susla GM, Kollef MH. 2004. Linezolid for treatment of ventilator-associated pneumonia: a cost-effective alternative to vancomycin. Crit Care Med 32: 137-143.

[62] Caisse nationale de l’Assurance maladie des travailleurs salariés. Classification commune des actes médicaux (CCAM): version 4 applicable au 17 mai 2006

[63] Physician survey; 2012, November

Appendix D

Table D.1 Characteristics of trials included in the network meta-analysis

| Study | cSSSI | HAP  /VAP | Sample size | Antibiotic group | Mean  age (years) | Male/Total (%) | Dosage regimen | | Primary outcome^1^ | Treatment duration (days) | Study design | Industry Sponsorship |
| --- | --- | --- | --- | --- | --- | --- | --- | --- | --- | --- | --- | --- |
| Wunderink  et al., 2012^[11]^ |  | √ | 348 | VAN | 61.6 | 112/176 (63.6) | | i.v. 15 mg/kg q12h | 81/174 | 7-30 | double-blind Phase 4 multinational | Pfizer |
|  |  |  |  | LIN | 60.7 | 116/172 (61.4) | | i.v. 600 mg q12h | 95/165 |  |  |  |
| Itani et al., 2010^[37]^ | √ |  | 1052 | VAN | 49.4 | 315/515 (61.0) | | i.v. 15 mg/kg q12h^a^ | 243/287 | 7-14 | open-label Phase 4 multicenter | Pfizer |
|  |  |  |  | LIN | 49.7 | 305/537 (57.0) | | i.v./p.o. 600 mg q12h | 254/284 |  |  |  |
| Lin et al., 2008^[14]^ | √ | √ | 142 | VAN | 59.6 | 46/71 (64.8) | | i.v. 1 g q12h | cSSSI:19/26  HAP:18/34 | 7-14 | double-blind Phase 3 multicenter | Pfizer |
|  |  |  |  | LIN | 56.3 | 42/71 (59.1) | | i.v. 600 mg q12h | cSSSI:31/33  HAP:31/33 |  |  |  |
| Wunderink et al., 2008^[38]^ |  | √ | 50 | VAN | 54.9 | 16/20 (80.0) | | i.v. 1 g q12h | 9/20 | 7-14 | open-label multicenter | Pfizer |
|  |  |  |  | LIN | 55.7 | 22/30 (73.3) | | i.v. 600 mg q12h | 13/30 |  |  |  |
| Kohno et al., 2007^[39]^ | √ | √ | 151 | VAN | 67.5 | 36/51 (70.6) | | i.v. 1 g q12h^b^ | cSSSI:6/10  HAP:9/19 | 7-21 | open-label multicenter | Pfizer |
|  |  |  |  | LIN | 68.4 | 70/100 (70.0) | | i.v. 600 mg q12h | cSSSI: 14/18  HAP:21/35 |  |  |  |
| Jaksic et al., 2006^[40]^ |  | √ | 605 | VAN | 48.1 | 161/301 (53.5) | | i.v. 1 g q12h | 45/50 | 7-14 | double-blind multinational | Pfizer |
|  |  |  |  | LIN | 47.2 | 179/304 (58.9) | | i.v. 600 mg q12h | 55/63 |  |  |  |
| Weigelt et al., 2005^[41]^ | √ |  | 1180 | VAN | 52 | 363/588 (61.7) | | i.v. 1 g q12h^c^ | 102/185 | 7-14 | open-label multicenter | Pfizer |
|  |  |  |  | LIN | 52 | 375/592 (63.3) | | i.v. 600 mg q12h | 125/176 |  |  |  |
| Wunderink et al., 2003^[42]^ |  | √ | 623 | VAN | 61.9 | 187/302 (61.9) | | i.v. 1 g q12h | 128/245 | 7-21 | double-blind multinational | Pfizer |
|  |  |  |  | LIN | 63.1 | 209/321 (65.1) | | i.v. 600 mg q12h | 135/256 |  |  |  |
| Stevens et al., 2002^[43]^ | √ | √ | 460 | VAN | 59.8 | 131/220 (59.5) | | i.v. 1 g q12h^b^ | cSSSI:54/87  HAP:16/32 | 7-14 | open-label multicenter | Pfizer |
|  |  |  |  | LIN | 63.9 | 143/240 (59.6) | | i.v. 600 mg q12h | cSSSI:64/99  HAP:20/39 |  |  |  |
| Rubinstein et al., 2001^[44]^ |  | √ | 396 | VAN | 61.3 | 131/193 (67.8) | | i.v. 1 g q12h^c^ | 62/91 | 7-20 | double-blind multinational | NR |
|  |  |  |  | LIN | 62.8 | 142/203 (70.0) | | i.v. 600 mg q12h | 71/107 |  |  |  |
| Dryden et al., 2016^[27]^ | √ |  | 761 | VAN/AZT | 53.6 | 148/255 (58.0) | | i.v. 15 mg/kg q12ha/i.v.  1 g q8h | 12/15 | 5-14 | double-blind Phase 3 multicenter | AstraZeneca |
|  |  |  |  | CEF^1^ | 52.6 | 310/506 (61.3) | | i.v. 600 mg q8h | 21/25 |  |  |  |
| Wilcox et al., 2010^[7]^ | √ |  | 680 | VAN | 47.5 | 201/338 (59.5) | | i.v. 1 g q12h | 289/338 | 5-14 | double-blind Phase 3 multicenter | Forest Laboratories |
|  |  |  |  | CEF^1^ | 47.8 | 224/342 (65.5) | | i.v. 600 mg q12h/i.v. 400mg q12h^e^ | 291/342 |  |  |  |
| Corey et al., 2010^[45]^ | √ |  | 698 | VAN | 49.2 | 218/347 (62.8) | | i.v. 1 g q12h | 297/347 | 5-14 | double-blind Phase 3 multicenter | Forest Laboratories |
|  |  |  |  | CEF^1^ | 47.2 | 220/351 (62.7) | | i.v. 600 mg q12h | 304/351 |  |  |  |
| Talbot et al., 2007^[46]^ | √ |  | 100 | VAN | 44.0 | 19/33 (59.4) | | i.v. 1 g q12h | 24/27 | 7-14 | observer-blinded Phase 2 multicenter | Cerexa |
|  |  |  |  | CEF^1^ | 41.6 | 37/67 (55.2) | | i.v. 600 mg q12h | 59/61 |  |  |  |
| Awad et al., 2014^[47]^ |  | √ | 571 | LIN | 65 | 170/284 (60.0) | | i.v. 600 mg q12h | 19/32 | 7-14 | double-blind Phase 3 multinational | Basilea Pharmaceutica |
|  |  |  |  | CEF^2^ | 65 | 202/287 (70.0) | | i.v. 500 mg q8h | 22/28 |  |  |  |
| Noel et al., 2008^[48]^ | √ |  | 784 | VAN | 46.7 | 236/387(61.0) | | i.v. 1 g q12h | 31/36 | 7-14 | double-blind multicenter | Johnson |
|  |  |  |  | CEF^2^ | 48 | 218/397(55.0) | | i.v. 500 mg q8h | 78/87 |  |  |  |
| Noel et al., 2008^[49]^ | √ |  | 543 | VAN | 46.7 | 219/267(82.0) | | i.v. 1 g q12h | 54/60 | 7-14 | double-blind multicenter | Johnson |
|  |  |  |  | CEF^2^ | 48 | 225/276(81.5) | | i.v. 500 mg q8h | 56/61 |  |  |  |
| Harbarth et al., 2015^[3]^ | √ | √ | 150 | SXT/RIF | 67 | 52/75(69.3) | | i.v./p.o. Trimethoprim  160 mg/sulfamethoxazole 800 mg q8h/rifampicin 600 mg q24h | cSSSI:16/21  HAP:6/8 | 7-14 | single-center open-label randomized | NR |
|  |  |  |  | LIN | 69 | 50/75(66.7) | | i.v./p.o. 600 mg q12h | cSSSI:20/24  HAP:5/9 |  |  |  |
| Ralph Corey et al., 2015^[26]^ | √^*^ |  | 1005 | VAN | 44.4 | 343/502(68.3) | | i.v.1 g or 15 mg/kg q12h | 82/101 | 7-10 | double-blind Phase 3 multicenter | Medicines Company |
|  |  |  |  | ORI | 45 | 338/503(67.2) | | i.v.1200 mg infused over 3 hours | 82/100 |  |  |  |
| Corey et al., 2014^[5]^ | √^*^ |  | 954 | VAN | 44.3 | 301/479(62.8) | | i.v. 1 g or 15 mg/kg q12h | 378/479 | 7-10 | double-blind multicenter | Medicines Company |
|  |  |  |  | ORI | 46.2 | 301/475(63.4) | | i.v. 1200 mg | 391/475 |  |  |  |
| Boucher et al., 2014 discovery 1^[6]^ | √ |  | 573 | VAN | 48.9 | 173/285(60.7) | | i.v. 1 g q12h | 233/285 | 10-14 | double-blind multicenter | Durata Therapeutics |
|  |  |  |  | DAL | 48.8 | 170/288(59.0) | | i.v. 1000 mg day1+500 mg day8 | 240/288 |  |  |  |
| Boucher et al., 2014 discovery 2^[6]^ | √ |  | 739 | VAN | 51.4 | 201/371(54.6) | | i.v. 1 g q12h | 288/368 | 10-14 | double-blind multicenter | Durata Therapeutics |
|  |  |  |  | DAL | 49.1 | 223/368(60.1) | | i.v. 1000 mg day1+500 mg day8 | 285/371 |  |  |  |
| Jauregui et al., 2005^[50]^ | √ |  | 854 | LIN | 46 | 172/283(61.0) | | i.v./p.o. 600 mg q12h | 85/97 | 7-14 | double-blind Phase 3 multicenter | Vicuron |
|  |  |  |  | DAL | 47 | 353/571(62.0) | | i.v. 1000 mg day1 +500 mg day8 | 162/181 |  |  |  |
| Aikawa et al., 2013^[25]^ | √ |  | 110 | VAN | 70 | 15/22(68.2) | | i.v. 1g q12h | 45/55 | 7-14 | open-label phase 3 multicenter | NR |
|  |  |  |  | DAP | 69 | 47/88(53.4) | | i.v. 4 mg/kg q24h | 16/19 |  |  |  |
| Pertel et al., 2009^[4]^ | √ |  | 101 | VAN | 55 | 25/51(49.0) | | i.v. 1 g q12h | 16/22 | 7-14 | evaluator-blinded multicenter | Cubist |
|  |  |  |  | DAP | 57 | 17/50(34.0) | | i.v. 4 mg/kg q24h | 22/28 |  |  |  |
| Katz et al., 2008^[13]^ | √ |  | 100 | VAN | 41 | 35/48(72.9) | | i.v. 1 g q12h | 31/35 | 7-14 | pilot study semi-single blind multicenter | Cubist |
|  |  |  |  | DAP | 43.5 | 31/38(64.6) | | i.v. 10 mg/kg q24h | 27/37 |  |  |  |
| Arbeit et al., 2004^[51]^ | √ |  | 899 | VAN | 51.5 | 295/471(55.0) | | i.v. 1 g q12h | 352/471 | 7-14 | multicenter evaluator-blinded | Cubist |
|  |  |  |  | DAP | 51.9 | 235/428(55.0) | | i.v. 10 mg/kg q24h | 318/428 |  |  |  |
| Stryjewski et al., 2012^[23]^ | √ |  | 152 | VAN | 40 | 59/76(60.0) | | i.v. 1 g q12h | 63/76 | 7-14 | double-blind Phase 2 multicenter | Theravance |
|  |  |  |  | TEL | 40 | 61/76(62.0) | | i.v. 2 mg/kg q24h | 62/76 |  |  |  |
| Rubinstein et al., 2011 ATTAIN 1/0015^[12]^ |  | √ | 746 | VAN | 62 | - | | i.v. 1 g q12h | 221/374 | 7-14 | double-blind Phase 3 multinational | Theravance |
|  |  |  |  | TEL | 63 | - | | i.v. 10 mg/kg q24h | 214/372 |  |  |  |
| Rubinstein et al., 2011 ATTAIN 2/0019^[12]^ |  | √ | 757 | VAN | 62 | - | | i.v. 1 g q12h | 227/377 | 7-14 | double-blind Phase 3 multinational | Theravance |
|  |  |  |  | TEL | 63 | - | | i.v. 10 mg/kg q24h | 228/380 |  |  |  |
| Stryjewski et al., 2008^[10]^ | √ |  | 1383 | VAN | 48.7 | 422/703(60.0) | | i.v. 1 g q12h | 260/301 | 10-14 | double-blind Phase 3 multicenter | Theravance |
|  |  |  |  | TEL | 48.8 | 380/680(56.0) | | i.v. 10 mg/kg q24h^c^ | 252/278 |  |  |  |
| Stryjewski et al., 2006^[52]^ | √ |  | 195 | VAN | 42.3 | 62/95(65.0) | | i.v. 1 g q12h^a^ | 81/95 | 4-14 | double-blind Phase 2 multicenter | Theravance |
|  |  |  |  | TEL | 44.7 | 55/100(55.0) | | i.v. 10 mg/kg q24h^e^ | 82/100 |  |  |  |
| Stryjewski et al., 2005^[53]^ | √ |  | 167 | VAN | 44.3 | 46/83(55.0) | | i.v. 1 g q12h^b^ | 66/83 | 7-14 | double-blind Phase 2 multicenter | Theravance |
|  |  |  |  | TEL | 44.6 | 54/84(64.0) | | i.v. 10 mg/kg q24h^e^ | 66/84 |  |  |  |
| Florescu et al., 2008^[2]^ | √ |  | 108 | VAN | 51 | 16/27(59.0) | | i.v. 1 g q12h^c^ | 20/23 | 7-28 | double-blind Phase 3 multicenter | Wyeth |
|  |  |  |  | TIG | 51 | 52/81(65.0) | | I v. 100 mg+(50 mg q12h) | 51/59 |  |  |  |
| Sacchidanand et al., 2005^[54]^ | √ |  | 573 | VAN/AZT | 48.4 | 188/281(66.9) | | i.v. Vancomycin 1 g+ aztreonam 1 g q12h | 118/141 | up to 14 | double-blind Phase 3 multicenter | NR |
|  |  |  |  | TIG | 49.4 | 180/292(61.6) | | i.v. 100 mg+(50 mg q12h) | 109/133 |  |  |  |
| Breedt et al., 2005^[24]^ | √ |  | 543 | VAN/AZT | 50.1 | 167/269(60.9) | | i.v. Vancomycin 1 g+ aztreonam 1 g q12h | 225/259 | up to 14 | double-blind Phase 3 multicenter | Wyeth |
|  |  |  |  | TIG | 48.8 | 163/274(60.6) | | i.v. 100 mg+(50 mg q12h) | 220/261 |  |  |  |
| Cepeda et al., 2004^[55]^ |  | √ | 204 | LIN | 59.2 | 67/100(67.0) | | i.v. 600 mg q12h | 67/71 | 7-21 | double-blind multinational | Pfizer |
|  |  |  |  | TEI | 57.3 | 68/104(65.4) | | i.v. 400 mg q12h for three doses then 400 mg q24h | 90/92 |  |  |  |
| Wilcox et al., 2004^[8]^ | √ |  | 430 | LIN | 53 | 117/215(54.0) | | i.v. 600 mg q12h | 113/117 | 7-28 | open-label Phase 3 multicenter | Pfizer |
|  |  |  |  | TEI | 55 | 117/215(54.0) | | i.v. at the discretion of the study investigator | 103/111 |  |  |  |
| Noel et al., 2012^[56]^ | √ |  | 219 | LIN | 50 | 57/108(53.0) | | i.v./p.o. 600 mg q12h | 59/75 | 7-10 | evaluator-blind phase 2 multicenter | Paratek |
|  |  |  |  | OMA | 51 | 66/111(59.0) | | i.v. 100 mg q24h/p.o. 200 mg q24h | 78/84 |  |  |  |
| Craft et al., 2011^[57]^ | √^*^ |  | 156 | LIN | 40.6 | 50/77(56.0) | | p.o. 600 mg q12h | 73/77 | 10-14 | double-blind Phase 2 multicenter | Cempra |
|  |  |  |  | CEM-102 | 41.5 | 56/78(72.0) | | p.o. 1500 mg q12h day 1+600 mg q12h | 67/78 |  |  |  |
| Covington et al., 2011^[58]^ | √ |  | 161 | LIN | 36.9 | - | | i.v. 600 mg q12h | 38/59 | 10-14 | double-blind Phase 2 multicenter | Johnson |
|  |  |  |  | JNJ-Q2 | 36.9 | - | | i.v. 250 mg q12h | 43/62 |  |  |  |
| Moran et al.,2014^[22]^ | √^*^ |  | 666 | LIN | 46 | 214/334(64.0) | | i.v. 600 mg q12h | 44/53 | 6-10 | double-blind Phase 3 multicenter | Cubist |
|  |  |  |  | TED | 46 | 225/332(68.0) | | i.v. 200 mg q24h | 44/56 |  |  |  |
| Prokocimer et al., 2013^[1]^ | √^*^ |  | 667 | LIN | 43.1 | 198/335(59.1) | | i.v. 600 mg q12h | 75/88 | 6-10 | double-blind Phase 3 multicenter | Trius Therapeutics |
|  |  |  |  | TED | 43.6 | 204/332(61.4) | | i.v. 200 mg q24h | 77/90 |  |  |  |
| Jung et al., 2010^[59]^ |  | √ | 83 | VAN | 71 | 35/41(85.3) | | i.v. 1 g q12h^c^ | 22/41 | 7-14 | open-label | NR |
|  |  |  |  | VAN/RIF | 66 | 35/42(83.3) | | i.v. 1 g q12h/p.o. 300 mg q12h | 13/42 |  |  |  |
| Fagon et al., 2000^[9]^ |  | √ | 298 | VAN | 56.6 | - | | i.v. 1 g q12h^c^ | 67/148 | 7-14 | open-label multicenter | NR |
|  |  |  |  | Q/D | 57.9 | - | | i.v. 7.5 mg/kg q8h | 65/150 |  |  |  |

cSSSI, complicated skin and soft structure infection. HAP/VAP, hospital-acquired or ventilator-associated pneumonia. mITT, modified intention-to-treat population. i.v., intravenous. p.o., oral. VAN, vancomycin. LIN, linezolid. VAN/AZT, vancomycin plus aztreonam. CEF^1^, ceftaroline. CEF^2^, ceftobiprole. SXT/RIT, trimethoprim/sulfamethoxazole plus rifampicin. ORI, oritavancin. DAL, dalbavancin. DAP, daptomycin. TEL, telavancin. TIG, tigecyline. TEI, teicoplanin. OMA, omadacycline. CEM-102, fusidic acid. JNJ-Q2, a novel fluoroquinolone. TED, tedizolid. VAN/RIF, vancomycin plus rifampicin. Q/D, quinupristin/dalfopristin. NR, not reported.

^1^ The data was shown as the events divided by the total numbers.

* ABSSSI, acute bacterial skin and skin structure infection.

^a^ Adjusted to trough concentration and creatinine clearance.

^b^ TDM, Therapeutic durg monitoring.

^c^ Adjusted to renal function

^e^ Dosage adjusted to renal function: 30 < creatinine clearance ≤ 50 mL/min

**Appendix E**

Figure E.1 Risk of bias graph in cSSSI


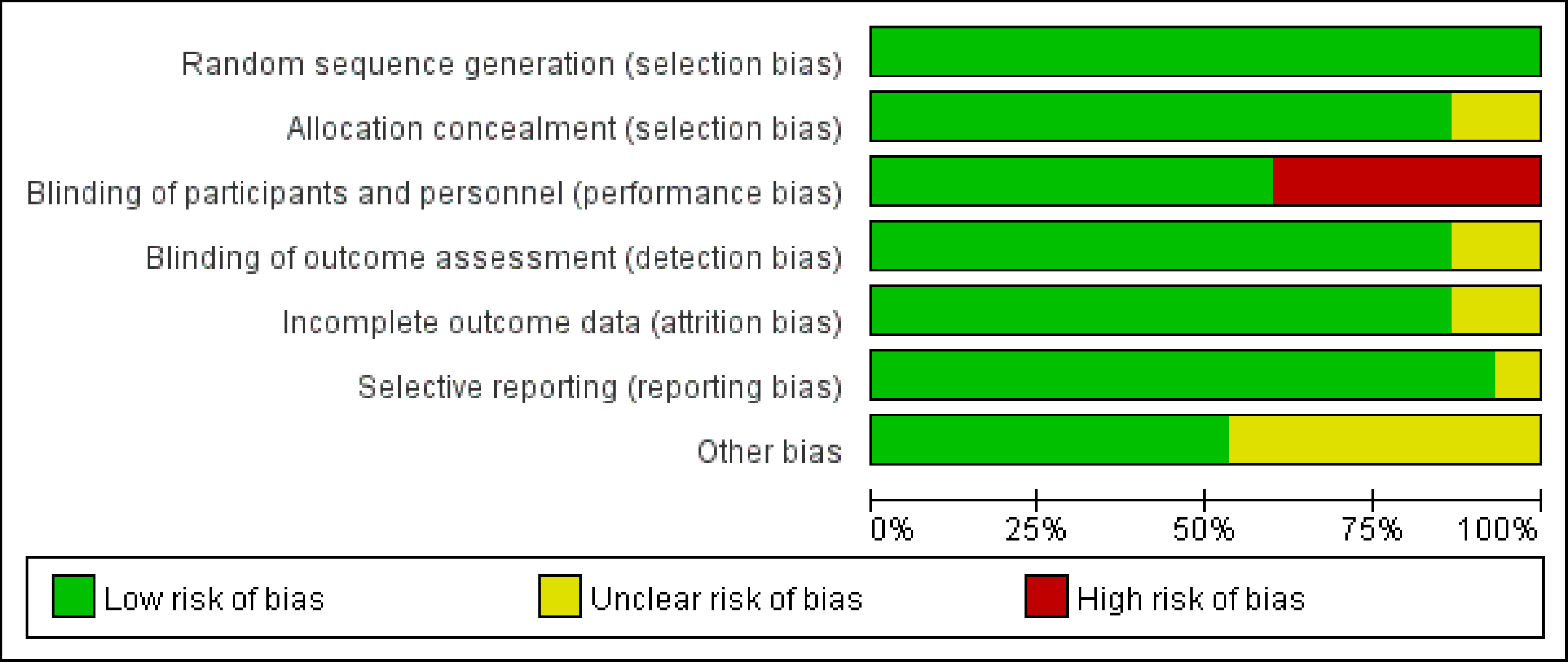


cSSSI, complicated skin and soft structure infection.

Figure E.2 Risk of bias summary in cSSSI


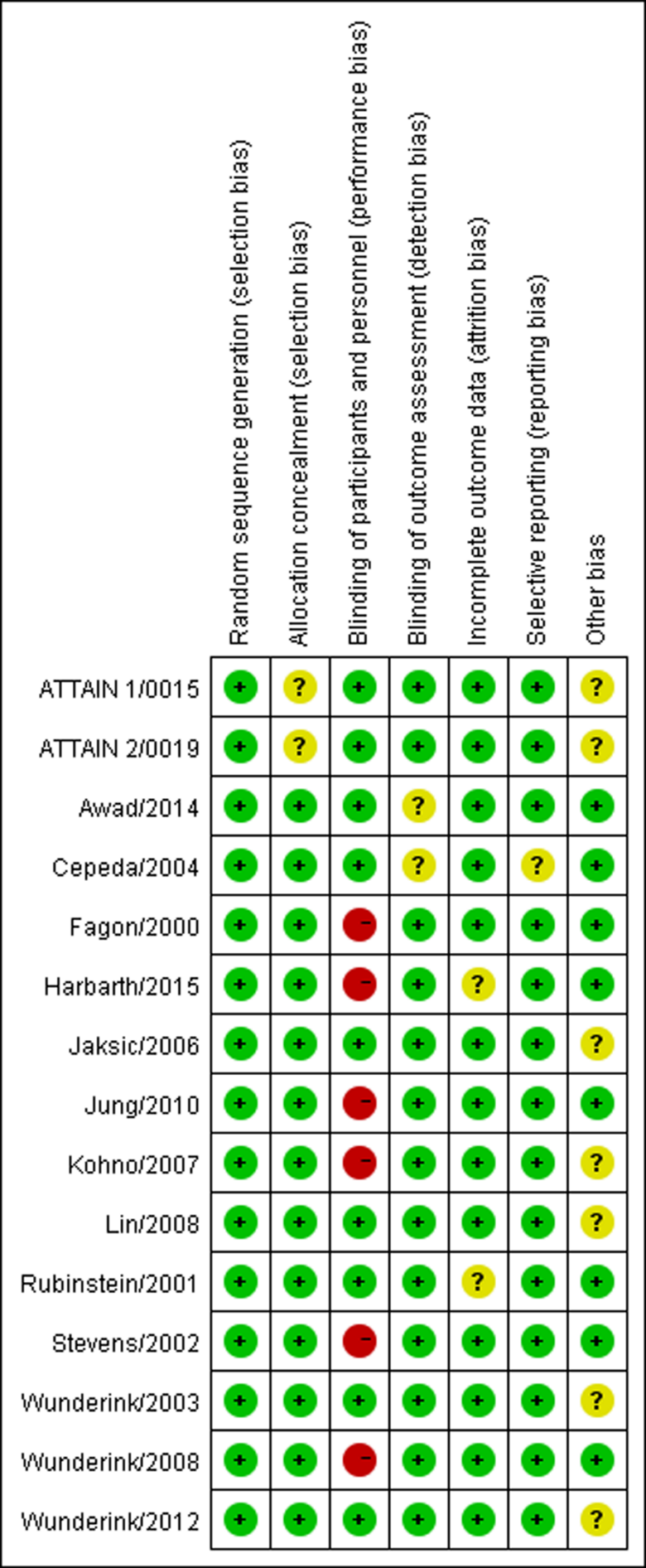


cSSSI, complicated skin and soft structure infection.

Figure E.3 Risk of bias graph in pneumonia


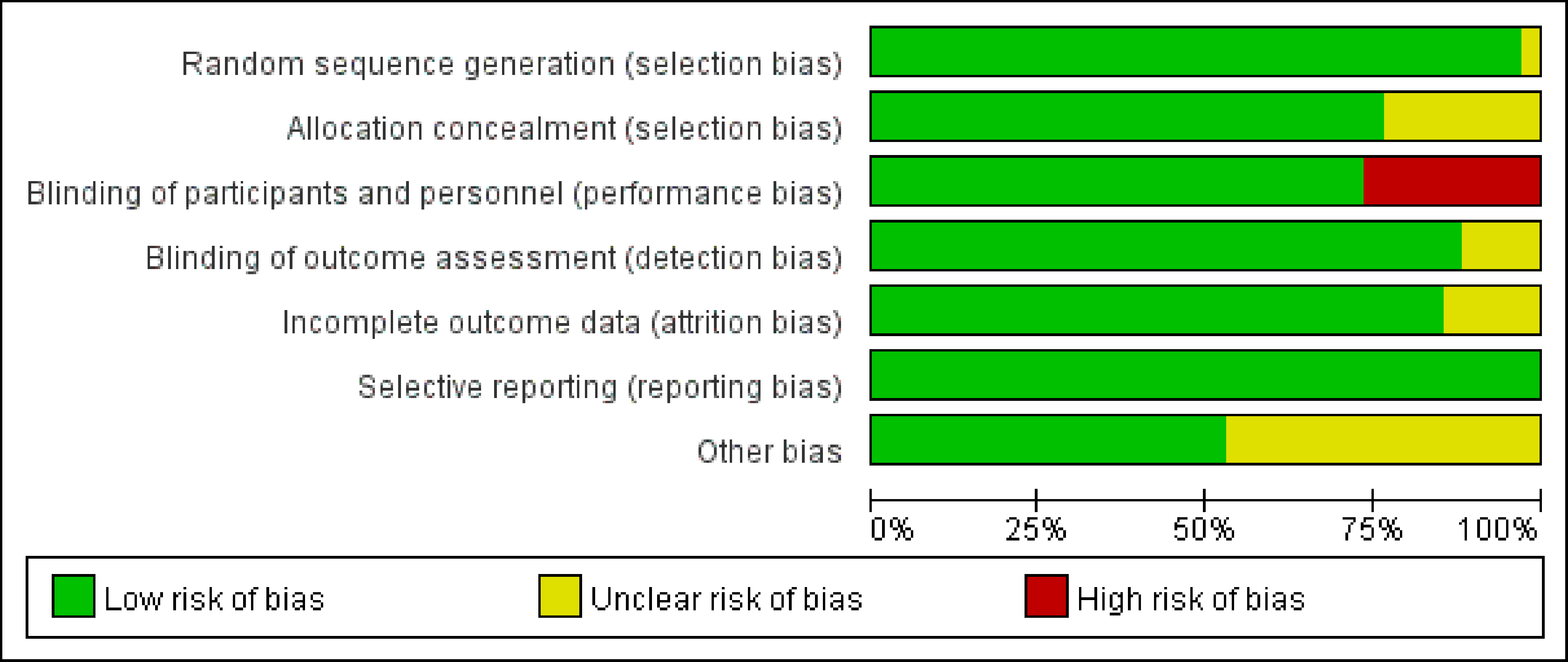


Figure E.4 Risk of bias summary in pneumonia


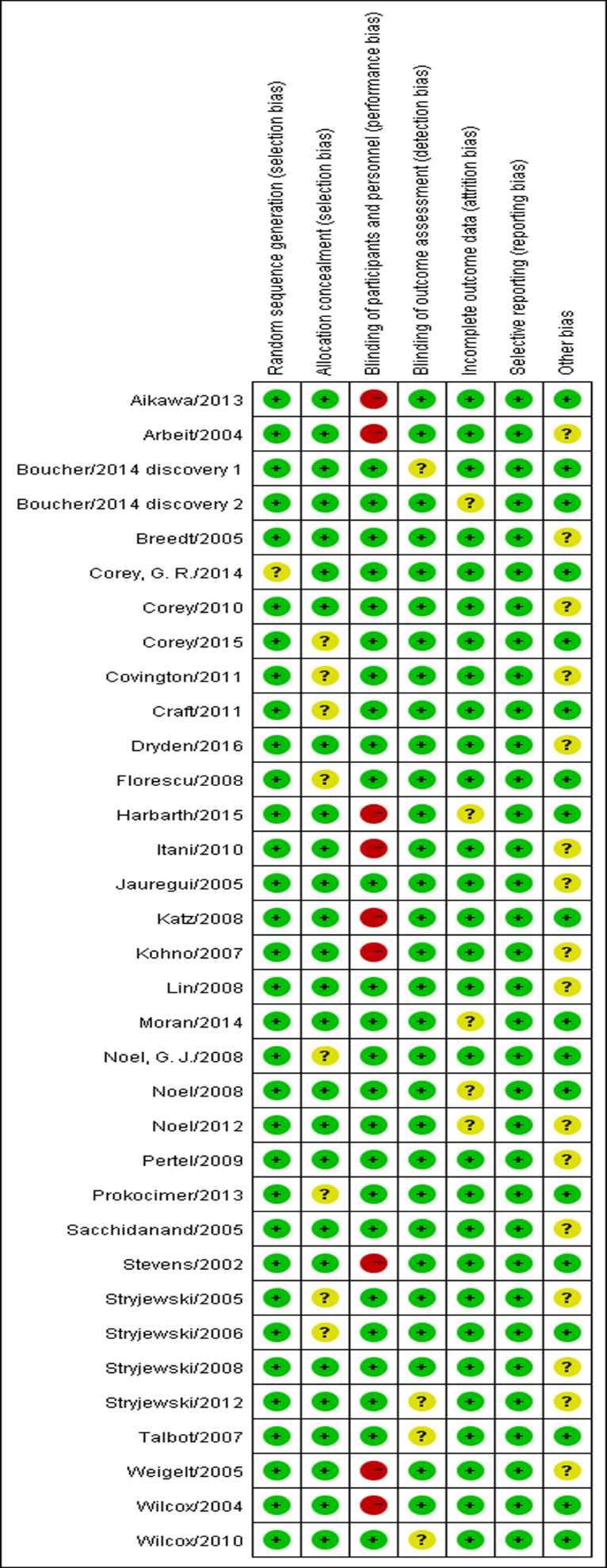


**Appendix F**

**Table F.1** Pairwise meta-analysis of antibiotics of clinical response in cSSSI in ME population and CE population

| Treatment comparison | Number of studies | Pairwise meta-analysis  Odds ratio (95% CI) | *P* value | Heterogeneity *I^2^*, % (variation in OR attributable to heterogeneity) |
| --- | --- | --- | --- | --- |
| Microbial clearance rates (ME) | | | |  |
| Vancomycin vs Daptomycin | 1 | 1.33(0.06, 26.62) | 0.851 | - |
| Vancomycin vs Telavancin | 4 | 0.74(0.39, 1.42) | 0.372 | 0.0 |
| Vancomycin vs Ceftaroline | 4 | 0.99(0.47, 2.09) | 0.983 | 0.0 |
| Vancomycin vs Tigecycline | 2 | 1.31(0.78, 2.17) | 0.300 | 0.0 |
| Linezolid vs Vancomycin | 2 | 1.93(1.26, 2.96) | 0.002 | 0.0 |
| Linezolid vs Fusidic Acid | 1 | 3.57(0.14, 90.78) | 0.441 | - |
| Linezolid vs Tedizolid | 1 | 0.81(0.33, 2.01) | 0.654 | - |
| Clinical cure rates (CE) | | | |  |
| Vancomycin vs Telavancin | 2 | 0.78(0.33, 1.83) | 0.566 | 0.0 |
| Vancomycin vs Tigecycline | 2 | 1.55(0.87, 2.81) | 0.140 | 0.0 |
| Vancomycin vs Ceftaroline | 4 | 0.76(0.30, 1.92) | 0.563 | 0.0 |
| Vancomycin vs Daptomycin | 2 | 1.13(0.83, 1.58) | 0.485 | 0.0 |
| Linezolid vs Tedizolid | 1 | 0.96(0.58, 1.57) | 0.880 | - |

cSSSI, complicated skin and soft structure infection. ME, microbiologically evaluable. CE, clinically evaluable. OR, odds ratio.

Figure F.1 Estimated OR (95%CI) values in ME population and in CE population of the network meta-analysis for cSSSI


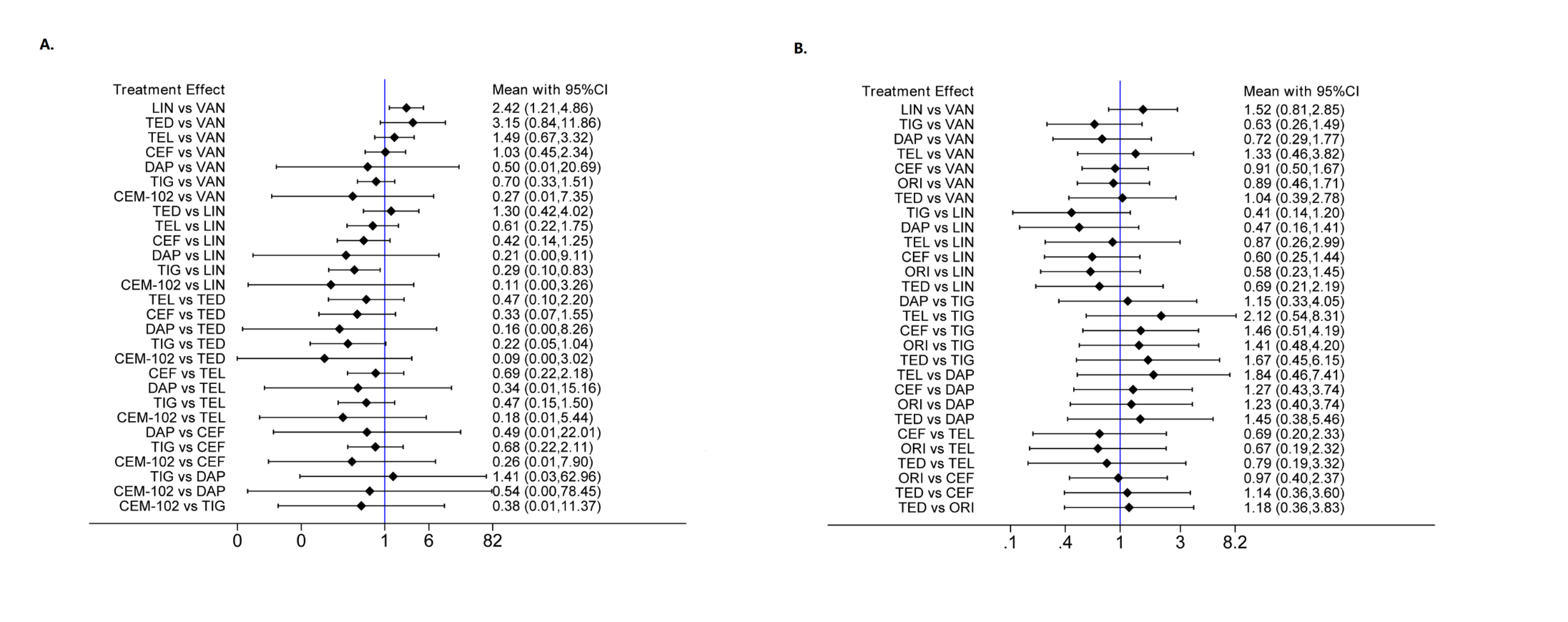


A. Estimated OR (95%CI) values for the microbiological response in ME population of the network meta-analysis. B. Estimated OR (95%CI) values for the clinical response in CE population of the network meta-analysis. OD, odds ratio. CI, confidence interval. ME, microbiologically evaluable. CE, clinically evaluable. cSSSI, complicated skin and soft structure infection. VAN, vancomycin. LIN, linezolid. CEF, ceftaroline. ORI, oritavancin. DAP, daptomycin. TEL, telavancin. TIG, tigecyline. CEM-102, fusidic acid. TED, tedizolid.

Figure F.2 Network meta-analysis of total adverse events in cSSSI in ITT population.


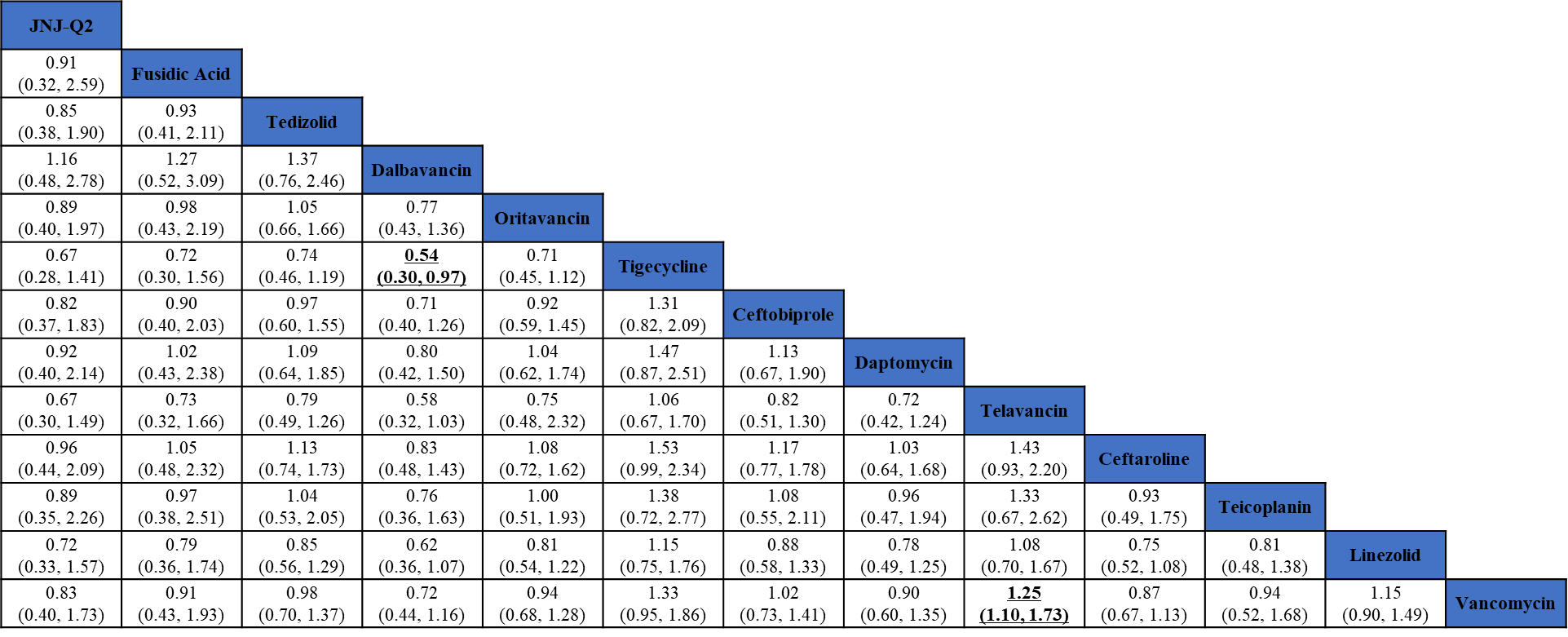
Comparisons should be read from left to right. The safety estimate is located at the intersection of the column-defining treatment and the row-defining treatment. Significant results are in bold and underlined. cSSSI, complicated skin and soft structure infection. ITT, intention-to-treat population. JNJ-Q2, a novel fluoroquinolone.

Table F.2 Pairwise meta-analysis of antibiotics of adverse events in cSSSI in ITT population.

| Treatment comparison | Number of studies | Odds ratio (95%CI) | *P* value |
| --- | --- | --- | --- |
| Total AEs | | | |
| Vancomycin vs Linezolid | 5 | 0.97 (0.86, 1.09) | 0.639 |
| Vancomycin vs Ceftaroline | 4 | 1.06 (0.92, 1.22) | 0.404 |
| Vancomycin vs Daptomycin | 3 | 1.04 (0.87, 1.23) | 0.656 |
| Vancomycin vs Ceftobiprole | 2 | 0.99 (0.83, 1.18) | 0.927 |
| Vancomycin vs Tigecycline | 3 | 0.84 (0.67, 1.05) | 0.125 |
| Vancomycin vs Oritavancin | 2 | 1.03 (0.88, 1.22) | 0.66 |
| Telavancin vs Vancomycin | 4 | 1.35 (1.14, 1.60) | <0.001 |
| Linezolid vs Dalbavancin | 1 | 1.10 (0.88, 1.37) | 0.402 |
| Linezolid vs Tedizolid | 2 | 1.01 (0.84, 1.21) | 0.931 |
| Linezolid vs Fusidic Acid | 1 | 1.06 (0.58, 1.93) | 0.85 |
| Linezolid vs JNJ-Q2 | 1 | 1.12 (0.62, 2.02) | 0.703 |
| Linezolid vs Teicoplanin | 1 | 1.11 (0.79, 1.56) | 0.519 |
| SAEs | | | |
| Vancomycin vs Linezolid | 3 | 1.00 (0.87, 1.14) | 0.995 |
| Vancomycin vs Tigecycline | 2 | 0.98 (0.79, 1.24) | 0.972 |
| Vancomycin vs Daptomycin | 2 | 1.02 (0.86, 1.20) | 0.833 |
| Vancomycin vs Ceftobiprole | 2 | 0.99 (0.86, 1.15) | 0.929 |
| Vancomycin vs Oritavancin | 2 | 1.00 (0.88, 1.13) | 0.994 |
| Vancomycin vs Ceftaroline | 1 | 1.01 (0.82, 1.25) | 0.916 |
| Vancomycin vs Telavancin | 2 | 1.03 (0.91, 1.17) | 0.637 |
| Linezolid vs Tedizolid | 2 | 0.99 (0.85, 1.16) | 0.983 |
| Linezolid vs Fusidic Acid | 1 | 1.04 (0.66, 1.63) | 0.864 |
| Nephrotoxicity | | | |
| Vancomycin vs Linezolid | 2 | 1.03 (0.82, 1.29) | 0.812 |
| Vancomycin vs Telavancin | 2 | 0.98 (0.86, 1.11) | 0.74 |
| Vancomycin vs Ceftobiprole | 1 | 1.01 (0.83, 1.25) | 0.893 |
| Vancomycin vs Daptomycin | 2 | 1.01 (0.86, 1.18) | 0.927 |
| Thrombocytopenia | | | |
| Vancomycin vs Linezolid | 3 | 0.95 (0.82, 1.10) | 0.508 |
| Linezolid vs Dalbavancin | 1 | 1.02 (0.84, 1.25) | 0.822 |
| Nausea | | | |
| Vancomycin vs Linezolid | 5 | 1.03 (0.93, 1.15) | 0.514 |
| Vancomycin vs Telavancin | 4 | 1.13 (1.00, 1.27) | 0.039 |
| Vancomycin vs Ceftobiprole | 2 | 1.06 (0.91, 1.22) | 0.451 |
| Vancomycin vs Ceftaroline | 4 | 1.01 (0.89, 1.14) | 0.911 |
| Vancomycin vs Daptomycin | 2 | 0.97 (0.82, 1.14) | 0.692 |
| Vancomycin vs Tigecycline | 3 | 0.81 (0.70, 0.91) | <0.001 |
| Vancomycin vs Oritavancin | 2 | 0.99 (0.87, 1.13) | 0.926 |
| Linezolid vs Tedizolid | 2 | 0.95 (0.82, 1.12) | 0.57 |
| Linezolid vs Omadacycline | 1 | 1.05 (0.71, 1.54) | 0.808 |
| Linezolid vs Fusidic Acid | 1 | 0.94 (0.58, 1.53) | 0.822 |
| Linezolid vs JNJ-Q2 | 1 | 1.15 (0.73, 1.82) | 0.552 |
| Linezolid vs Dalbavancin | 1 | 0.97 (0.79, 1.20) | 0.829 |
| Vomiting | | | |
| Vancomycin vs Linezolid | 5 | 0.98 (0.89, 1.09) | 0.81 |
| Vancomycin vs Telavancin | 4 | 0.94 (0.84, 1.05) | 0.309 |
| Vancomycin vs Ceftobiprole | 2 | 0.97 (0.84, 1.13) | 0.72 |
| Vancomycin vs Ceftaroline | 1 | 0.99 (0.80, 1.23) | 0.95 |
| Vancomycin vs Daptomycin | 2 | 1.01 (0.86, 1.18) | 0.93 |
| Vancomycin vs Tigecycline | 2 | 0.83 (0.70, 0.98) | 0.037 |
| Vancomycin vs Oritavancin | 2 | 1.00 (0.88, 1.14) | 0.99 |
| Linezolid vs Dalbavancin | 1 | 0.99 (0.81, 1.21) | 0.932 |
| Linezolid vs Tedizolid | 2 | 1.03 (0.88, 1.20) | 0.718 |
| Linezolid vs Omadacycline | 1 | 0.99 (0.68, 1.45) | 0.966 |
| Linezolid vs Fusidic Acid | 1 | 0.96 (0.61, 1.52) | 0.858 |
| Linezolid vs JNJ-Q2 | 1 | 0.94 (0.60, 1.47) | 0.782 |
| Diarrhea | | | |
| Vancomycin vs Linezolid | 4 | 0.95 (0.86, 1.06) | 0.414 |
| Vancomycin vs Ceftaroline | 3 | 0.99 (0.87, 1.12) | 0.883 |
| Vancomycin vs Telavancin | 3 | 1.01 (0.89, 1.13) | 0.893 |
| Vancomycin vs Tigecycline | 2 | 0.96 (0.81, 1.14) | 0.675 |
| Vancomycin vs Daptomycin | 2 | 0.99 (0.84, 1.17) | 0.953 |
| Vancomycin vs Oritavancin | 2 | 0.99 (0.87, 1.13) | 0.943 |
| Vancomycin vs Ceftobiprole | 2 | 0.98 (0.85, 1.14) | 0.833 |
| Linezolid vs Dalbavancin | 1 | 1.03 (0.84, 1.27) | 0.748 |
| Linezolid vs Tedizolid | 2 | 1.01 (0.87, 1.18) | 0.857 |
| Linezolid vs Omadacycline | 1 | 1.03 (0.70, 1.50) | 0.878 |
| Discontinuation | | | |
| Vancomycin vs Linezolid | 3 | 1.00 (0.87, 1.14) | 0.991 |
| Vancomycin vs Telavancin | 4 | 1.01 (0.90, 1.14) | 0.811 |
| Vancomycin vs Tigecycline | 3 | 0.99 (0.85, 1.17) | 0.972 |
| Vancomycin vs Daptomycin | 1 | 0.99 (0.84, 1.18) | 0.977 |
| Vancomycin vs Ceftaroline | 3 | 0.99 (0.87, 1.13) | 0.928 |
| Vancomycin vs Oritavancin | 2 | 0.99 (0.87, 1.13) | 0.938 |
| Vancomycin vs Ceftobiprole | 2 | 0.99 (0.86, 1.15) | 0.945 |
| Linezolid vs Dalbavancin | 1 | 1.00 (0.82, 1.23) | 0.946 |
| Linezolid vs Tedizolid | 2 | 0.99 (0.85, 1.16) | 0.953 |
| Linezolid vs Teicoplanin | 1 | 0.99 (0.75, 1.29) | 0.944 |
| All-cause mortality | | | |
| Vancomycin vs Linezolid | 5 | 1.00 (0.84, 1.19) | 0.898 |
| Vancomycin vs Ceftaroline | 1 | 0.99 (0.86, 1.15) | 0.956 |
| Vancomycin vs Telavancin | 1 | 1.00 (0.88, 1.14) | 0.999 |
| Vancomycin vs Tigecycline | 3 | 0.99 (0.85, 1.16) | 0.953 |
| Vancomycin vs Daptomycin | 2 | 1.00 (0.85, 1.18) | 0.959 |
| Vancomycin vs Oritavancin | 1 | 1.00 (0.89, 1.10) | 0.982 |
| Vancomycin vs Ceftobiprole | 2 | 1.00 (0.87, 1.15) | 0.981 |
| Linezolid vs Dalbavancin | 1 | 1.00 (0.82, 1.22) | 0.972 |
| Linezolid vs Teicoplanin | 1 | 0.98 (0.75, 1.29) | 0.914 |
| Linezolid vs Tedizolid | 1 | 1.00 (0.86, 1.16) | 0.972 |
| Linezolid vs SXT/rifampicin | 1 | 1.03 (0.65, 1.64) | 0.901 |

cSSSI, complicated skin and soft structure infection. ITT, intention-to-treat population. CI, confidence interval. AEs, adverse events. SAEs, serious adverse events. JNJ-Q2, a novel fluoroquinolone. SXT, trimethoprim/sulfamethoxazole.

Table F.3 Results of SUCRA in clinical cure and sensitivity analyses network meta-analysis for cSSSI

| Treatment | Standard analysis (All RCTs) | SUCRA rank | Excluding high risk studies | SUCRA rank | Adjusted for  sex ratio | SUCRA rank | Adjusted for  mean age | SUCRA rank |
| --- | --- | --- | --- | --- | --- | --- | --- | --- |
| Omadacycline | 0.09(0.02, 0.45) | 1 | 0.09(0.02, 0.45) | - | 0.12(0.02, 0.60) | 1 | 0.14(0.04, 0.49) | 1 |
| SXT/rifampicin | 0.21(0.03, 1.42) | 2 | - | - | 0.25(0.04, 1.76) | 2 | 0.31(0.07, 1.67) | 2 |
| JNJ-Q2 | 0.26(0.04, 1.33) | 3 | 0.25(0.08, 1.45) | - | 0.31(0.07, 1.35) | 3 | 0.36(0.10, 1.89) | 3 |
| Linezolid | 0.33(0.10, 1.10) | 4 | 0.33(0.10, 1.10) | 1 | 0.32(0.10, 1.07) | 4 | 0.49(0.24, 1.01) | 4 |
| Tedizolid | 0.37(0.11, 1.46) | 5 | 0.37(0.10, 1.43) | 2 | 0.35(0.09, 1.56) | 5 | 0.51(0.17, 1.56) | 5 |
| Oritavancin | 0.43(0.12, 1.53) | 6 | 0.39(0.11, 1.43) | 3 | 0.50(0.10, 9.10) | 9 | 0.54(0.04, 1.39) | 6 |
| Telavancin | 0.47(0.13, 1.65) | 7 | 0.43(0.12, 1.57) | 4 | 0.43(0.12, 1.55) | 6 | 0.59(0.21, 1.40) | 7 |
| Ceftaroline | 0.49(0.14, 1.70) | 8 | 0.44(0.12, 1.62) | 5 | 0.47(0.13, 1.65) | 8 | 0.71(0.17, 3.00) | 8 |
| Dalbavancin | 0.49(0.14, 1.68) | 9 | 0.45(0.13, 1.60) | 6 | 0.46(0.13, 1.56) | 7 | 0.74(0.21, 2.20) | 9 |
| Vancomycin | 0.52(0.15, 1.76) | 10 | 0.47(0.13, 1.67) | 7 | 0.64(0.17, 2.45) | 10 | 0.76(0.62, 2.79) | 10 |
| Ceftobiprole | 0.56(0.13, 2.48) | 11 | 0.51(0.11, 2.34) | 8 | 0.83(0.05, 14.33) | 12 | 0.78(0.33, 1.83) | 11 |
| Daptomycin | 0.58(0.16, 2.20) | 12 | 0.54(0.16, 2.11) | 9 | 0.70(0.19, 2.54) | 11 | 0.88(0.42, 3.06) | 12 |
| Tigecycline | 0.61(0.17, 2.20) | 13 | 0.56(0.15, 2.08) | 10 | 0.87(0.94, 1.95) | 13 | 0.88(0.21, 3.76) | 13 |
| Teicoplanin | 0.73(0.20, 2.56) | 14 | - | - | 0.97(0.16, 6.61) | 14 | 0.95(0.26, 1.86) | 14 |
| Fusidic Acid | reference | 15 | reference | - | reference | 15 | reference | 15 |

Significant results are in bold and underlined. SUCRA, surface under the cumulative ranking curve. cSSSI, complicated skin and soft structure infection. RCT, randomized controlled trial. SXT, trimethoprim/sulfamethoxazole. JNJ-Q2, a novel fluoroquinolone.

Table F.4 Results of SUCRA in safety and sensitivity analyses network meta-analysis for cSSSI.

| Treatment | Standard analysis (All RCTs) | SUCRA rank | Excluding studies (without only one RCT) | SUCRA rank |
| --- | --- | --- | --- | --- |
| Dalbavancin | 0.54(0.30, 0.97) | 1 | - | - |
| Ceftaroline | 1.53(0.99, 2.34) | 2 | 0.65(0.31, 1.22) | 1 |
| JNJ-Q2 | 0.67(0.28, 1.41) | 3 | - | - |
| Daptomycin | 0.68(0.32, 1.33) | 4 | 0.68(0.32, 1.33) | 2 |
| Oritavancin | 0.71(0.45, 1.12) | 5 | 0.71(0.46, 1.14) | 3 |
| Fusidic Acid | 0.72(0.30, 1.56) | 6 | - | - |
| Teicoplanin | 0.72(0.46, 1.48) | 7 | - | - |
| Tedizolid | 0.74(0.46, 1.19) | 8 | 0.74(0.44, 1.21) | 4 |
| Vancomycin | 0.75(0.35, 1.26) | 9 | 0.75(0.35, 1.27) | 5 |
| Ceftobiprole | 0.76(0.28, 2.09) | 10 | 0.76(0.30, 2.09) | 6 |
| Linezolid | 0.87(0.51, 1.56) | 11 | 0.87(0.52, 1.56) | 7 |
| Tigecycline | 0.94(0.45, 1.65) | 12 | 0.94(0.45, 1.66) | 8 |
| Telavancin | reference | 13 | reference | 9 |

Significant results are in bold and underlined. SUCRA, surface under the cumulative ranking curve. cSSSI, complicated skin and soft structure infection.

RCT, randomized controlled trial. JNJ-Q2, a novel fluoroquinolone.

**Appendix G**

Table G.1 Pairwise meta-analysis of antibiotics of clinical response in pneumonia in ME population

| Treatment comparison | Number of studies | pairwise meta-analysis  Odds ratio (95%CI) | *P* value | Heterogeneity *I^2^*, % (variation in OR attributable to heterogeneity) |
| --- | --- | --- | --- | --- |
| Microbial clearance rates (ME) | | | | |
| Vancomycin vs Telavancin | 1 | 0.99(0.59-1.65) | 0.982 | - |
| Vancomycin vs Quinupristin/dalfopristin | 1 | 1.26(0.45-3.53) | 0.661 | - |
| Linezolid vs Vancomycin | 7 | 1.18(0.90-1.55) | 0.225 | 0.0 |
| Linezolid vs Ceftobiprole | 1 | 1.21(0.47-3.08) | 0.69 | - |
| Linezolid vs Teicoplanin | 1 | 1.13(0.57-2.22) | 0.729 | - |

ME, microbiologically evaluable. CI, confidence interval. OR, odds ratio.

Figure G.1 Network meta-analysis of total adverse events in pneumonia in ITT population


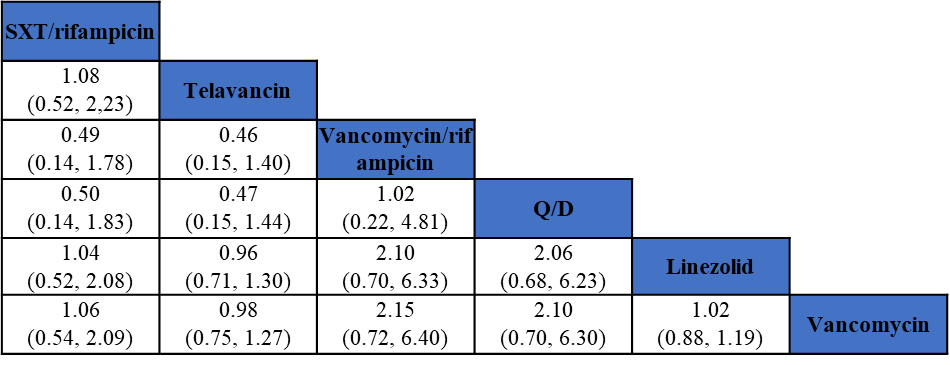


Comparisons should be read from left to right. The safety estimate is located at the intersection of the column-defining treatment and the row-defining treatment.

Significant results are in bold and underlined. ITT, intention-to-treat population. SXT, trimethoprim/sulfamethoxazole. Q/D, quinupristin/dalfopristin.

Table G.2 Pairwise meta-analysis of antibiotics of adverse events in pneumonia in ITT population

| Treatment comparison | Number of studies | Odds ratio (95% CI) | P value |
| --- | --- | --- | --- |
| Total AEs | | | |
| Vancomycin vs Linezolid | 7 | 0.99 (0.87, 1.12) | 0.885 |
| Vancomycin vs Quinupristin/dalfopristin | 1 | 0.49 (0.16, 1.47) | 0.207 |
| Vancomycin vs Vancomycin/rifampicin | 1 | 0.87 (0.47, 1.60) | 0.659 |
| Vancomycin vs Telavancin | 1 | 1.01 (0.78, 1.31) | 0.903 |
| Linezolid vs SXT/rifampicin | 1 | 0.96 (0.51, 1.81) | 0.904 |
| SAEs | | | |
| Vancomycin vs Linezolid | 5 | 1.01 (0.87, 1.16) | 0.881 |
| Vancomycin vs Quinupristin/dalfopristin | 1 | 0.94 (0.67, 1.33) | 0.747 |
| Vancomycin vs Telavancin | 1 | 0.93 (0.79, 1.09) | 0.384 |
| Linezolid vs SXT/rifampicin | 1 | 1.00 (0.61, 1.64) | 0.764 |
| Nausea | | | |
| Vancomycin vs Linezolid | 3 | 0.98 (0.82, 1.19) | 0.903 |
| Vancomycin vs Telavancin | 1 | 0.98 (0.85, 1.14) | 0.864 |
| Vomiting | | | |
| Vancomycin vs Linezolid | 3 | 0.98 (0.82, 1.18) | 0.878 |
| Discontinuation | | | |
| Vancomycin vs Linezolid | 7 | 1.01 (0.91, 1.12) | 0.822 |
| Vancomycin vs Quinupristin/dalfopristin | 1 | 0.93 (0.67, 1.30) | 0.692 |
| Vancomycin vs Telavancin | 1 | 0.97 (0.84, 1.12) | 0.7 |
| Diarrhea | | | |
| Vancomycin vs Linezolid | 3 | 0.99 (0.84-1.17) | 0.935 |
| Vancomycin vs Telavancin | 1 | 1.01 (0.87-1.17) | 0.89 |
| Linezolid vs Teicoplanin | 1 | 1.02 (0.69-1.52) | 0.908 |
| Nephrotoxicity | | | |
| Vancomycin vs Linezolid | 8 | 1.02 (0.93-1.12) | 0.661 |
| Vancomycin vs Vancomycin/rifampicin | 1 | 0.97 (0.54-1.75) | 0.933 |
| Vancomycin vs Telavancin | 1 | 0.97 (0.84-1.13) | 0.739 |
| Linezolid vs SXT/rifampicin | 1 | 0.98 (0.62-1.55) | 0.953 |
| Thrombocytopenia | | | |
| Vancomycin vs Linezolid | 3 | 0.99 (0.86-1.15) | 0.955 |
| Vancomycin vs Vancomycin/rifampicin | 1 | 1.04 (0.58-1.86) | 0.883 |
| Anemia | | | |
| Vancomycin vs Linezolid | 3 | 1.00 (0.87-1.16) | 0.933 |
| Vancomycin vs Telavancin | 1 | 0.99 (0.85-1.15) | 0.906 |
| All-cause mortality | | | |
| Vancomycin vs Linezolid | 7 | 1.01 (0.89, 1.15) | 0.998 |
| Vancomycin vs Quinupristin/dalfopristin | 1 | 0.59 (0.67, 1.34) | 0.783 |
| Vancomycin vs Vancomycin/rifampicin | 1 | 1.29 (0.66, 2.51) | 0.452 |
| Vancomycin vs Telavancin | 2 | 0.98 (0.84, 1,14) | 0.828 |
| Linezolid vs SXT/rifampicin | 1 | 1.03 (0.64, 1.64) | 0.901 |
| Linezolid vs Teicoplanin | 1 | 0.92 (0.61, 1.39) | 0.697 |
| Linezolid vs Ceftobiprole | 1 | 0.99 (0.80, 1.22) | 0.934 |

ITT, intention-to-treat population. CI, confidence interval. AEs, adverse events. SAEs, serious adverse events. SXT, trimethoprim/sulfamethoxazole.

Table G.3 Results of SUCRA in clinical cure and sensitivity analyses network meta-analysis for pneumonia

| Treatment | Standard analysis (All RCTs) | SUCRA rank | Excluding high risk studies | SUCRA rank | Adjusted for  sex ratio | SUCRA rank | Adjusted for  mean age | SUCRA rank |
| --- | --- | --- | --- | --- | --- | --- | --- | --- |
| Ceftobiprole | 0.13(0.03, 0.55) | 1 | 0.29(0.13, 3.14) | - | 0.13(0.03, 0.69) | 1 | 0.14(0.03, 0.70) | 1 |
| Teicoplanin | 0.18(0.01, 0.96) | 2 | 0.32(0.15, 5.26) | - | 0.14(0.02, 0.97) | 2 | 0.15(0.02, 0.96) | 2 |
| Linezolid | 0.32(0.13, 0.81) | 3 | 0.80(0.58, 1.09) | 1 | 0.32(0.12, 0.90) | 3 | 0.32(0.13, 0.82) | 3 |
| Vancomycin | 0.39(0.16, 0.95) | 4 | 0.96(0.78, 1.18) | 2 | 0.35(0.14, 1.03) | 4 | 0.37(0.90, 1.08) | 4 |
| Telavancin | 0.40(0.16, 1.01) | 5 | reference | 3 | 0.38(0.11, 1.28) | 5 | 0.42(0.15, 1.18) | 5 |
| Quinupristin/dalfopristin | 0.42(0.15, 1.15) | 6 | - | - | 0.42(0.13, 1.33) | 6 | 0.42(0.15, 1.16) | 6 |
| SXT/rifampicin | 0.77(0.08, 7.69) | 7 | - | - | 0.87(0.08, 9.09) | 7 | 0.80(0.07, 9.79) | 7 |
| Vancomycin/rifampicin | reference | 8 | - | - | reference | 8 | reference | 8 |

Significant results are in bold and underlined. SUCRA, surface under the cumulative ranking curve. RCT, randomized controlled trial. SXT, trimethoprim/sulfamethoxazole.

Table G.4 Results of SUCRA in safety and sensitivity analyses network meta-analysis for pneumonia

| Treatment | Standard analysis (All RCTs) | SUCRA rank | Excluding studies (without only one RCT) | SUCRA rank |
| --- | --- | --- | --- | --- |
| Vancomycin | 0.45(0.15, 1.38) | 1 | 0.98(0.84, 1.14) | 1 |
| Telavancin | 0.46(0.15, 1.40) | 2 | - | - |
| Linezolid | 0.47(0.16, 1.43) | 3 | reference | 2 |
| SXT/rifampicin | 0.49(0.14, 1.78) | 4 | - | - |
| Quinupristin/dalfopristin | 0.98(0.21, 4.75) | 5 | - | - |
| Vancomycin/rifampicin | reference | 6 | - | - |

SUCRA, surface under the cumulative ranking curve. RCT, randomized controlled trial. SXT, trimethoprim/sulfamethoxazole.

**Appendix H**

**Assessment of loop inconsistency in networks**

We estimated inconsistency as the difference between direct and indirect estimates (called inconsistency factor, IF) and the corresponding 95% confidence interval (CI) for each IF in each closed triangular or quadratic loop. The following graphs all show closed triangular loops (formed by three interventions) in each outcome network. Inconsistent loops are those that present inconsistency factors with 95% confidence intervals incompatible with zero.

Table H.1 Assessment of loop inconsistency in networks

| **Loop** | **IF (95% CI)** | **p value** | **Loop heterogeneity tau^2^** |
| --- | --- | --- | --- |
| Dalbavancin-Linezolid-Vancomycin | 0.551 (0.00, 1.40) | 0.204 | 0.000 |

IF, inconsistency factor. CI, confidence interval.

**Appendix I**

**Figure I.1 Comparison-adjusted funnel plot for cSSSI**


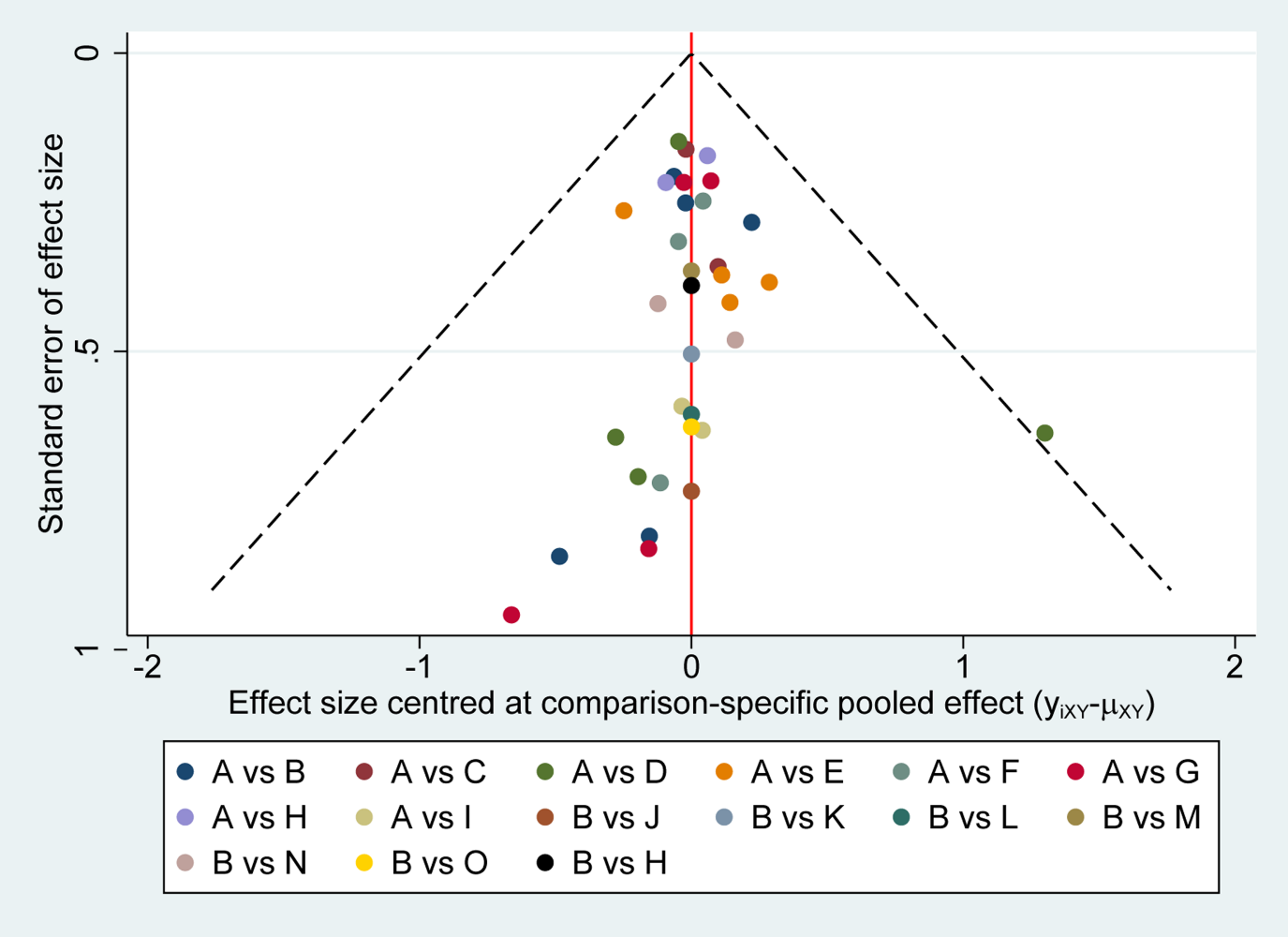


cSSSI, complicated skin and soft structure infections. A, Vancomycin. B, Linezolid. C, Oritavancin. D, Daptomycin. E, Telavancin. F, Tigecycline. G, Ceftaroline. H, Dalbavancin. I, Ceftobiprole. J, SXT/rifampicin. K, Omadacycline. L, Fusidic Acid. M, JNJ-Q2. N, Tedizolid. O, Teicoplanin.

**Figure I.2 Comparison-adjusted funnel plot for** pneumonia


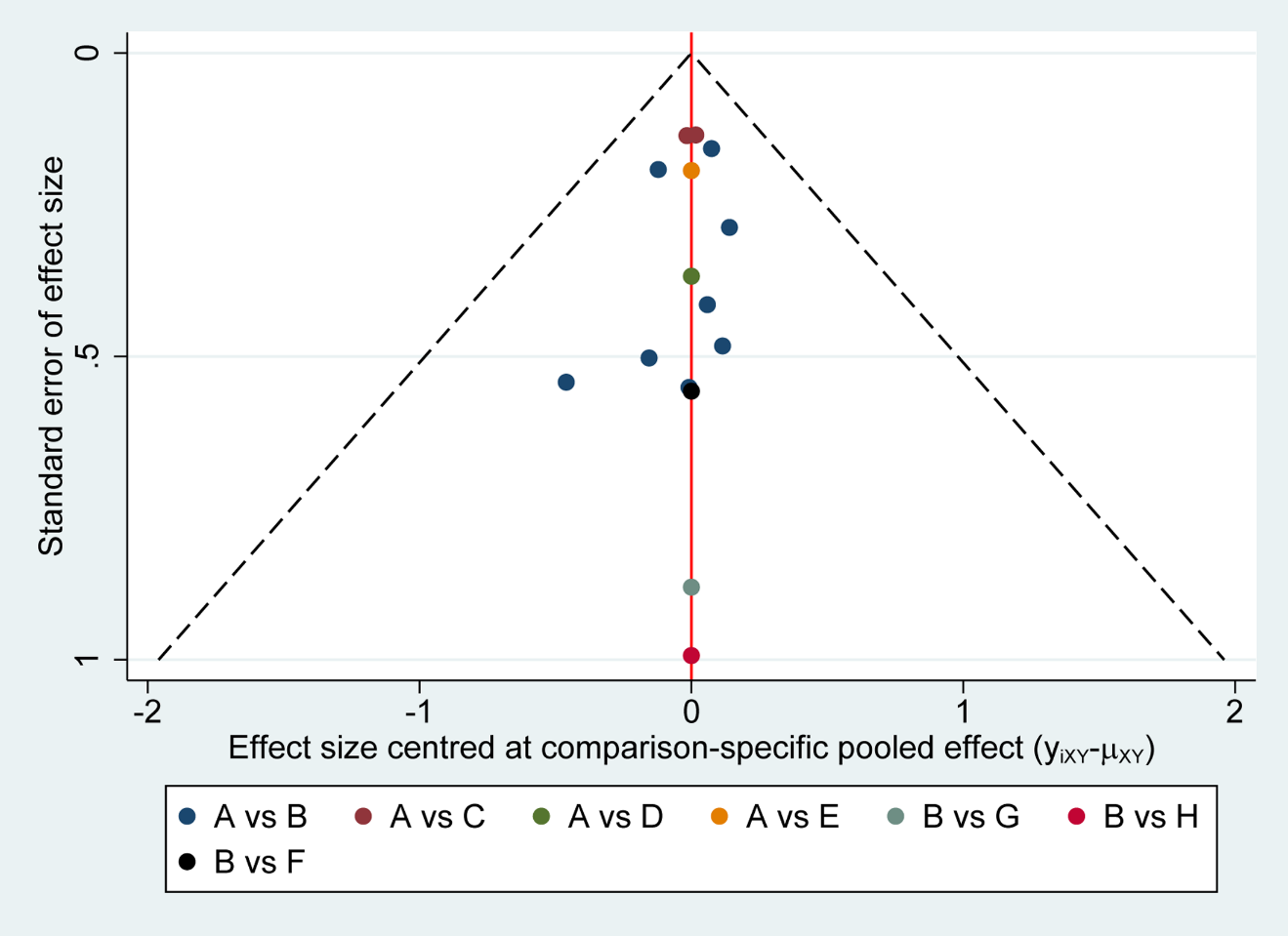


A, Vancomycin. B, Linezolid. C, Telavancin. D, Vancomycin/rifampicin. E, Quinupristin/dalfopristin. F, Ceftobiprole. G, Teicoplanin. H, SXT/rifampicin.
